# Molecular mapping and genomics of soybean seed protein: a review and perspective for the future

**DOI:** 10.1007/s00122-017-2955-8

**Published:** 2017-08-11

**Authors:** Gunvant Patil, Rouf Mian, Tri Vuong, Vince Pantalone, Qijian Song, Pengyin Chen, Grover J. Shannon, Tommy C. Carter, Henry T. Nguyen

**Affiliations:** 1 Division of Plant Sciences, University of Missouri, Columbia, MO 65211 USA; 2 Soybean and Nitrogen Fixation Unit, USDA-ARS, Raleigh, NC 27607 USA; 3 Department of Plant Sciences, University of Tennessee, Knoxville, TN 37996-4561 USA; 4 Agricultural Research Service, Department of Agriculture United States, Beltsville, MD 20705 USA

## Abstract

*****Key message***:**

**Genetic improvement of soybean protein meal is a complex process because of negative correlation with oil, yield, and temperature. This review describes the progress in mapping and genomics, identifies knowledge gaps, and highlights the need of integrated approaches.**

**Abstract:**

Meal protein derived from soybean [*Glycine max* (L) Merr.] seed is the primary source of protein in poultry and livestock feed. Protein is a key factor that determines the nutritional and economical value of soybean. Genetic improvement of soybean seed protein content is highly desirable, and major quantitative trait loci (QTL) for soybean protein have been detected and repeatedly mapped on chromosomes (Chr.) 20 (*LG*-*I*), and 15 (*LG*-*E*). However, practical breeding progress is challenging because of seed protein content’s negative genetic correlation with seed yield, other seed components such as oil and sucrose, and interaction with environmental effects such as temperature during seed development. In this review, we discuss rate-limiting factors related to soybean protein content and nutritional quality, and potential control factors regulating seed storage protein. In addition, we describe advances in next-generation sequencing technologies for precise detection of natural variants and their integration with conventional and high-throughput genotyping technologies. A syntenic analysis of QTL on Chr. 15 and 20 was performed. Finally, we discuss comprehensive approaches for integrating protein and amino acid QTL, genome-wide association studies, whole-genome resequencing, and transcriptome data to accelerate identification of genomic hot spots for allele introgression and soybean meal protein improvement.

**Electronic supplementary material:**

The online version of this article (doi:10.1007/s00122-017-2955-8) contains supplementary material, which is available to authorized users.

## Introduction

A major portion of human, poultry, and livestock diets around the globe is derived from cereals and legumes (Mandal and Mandal 2000). Soybean is considered a unique leguminous crop, because its seed is a rich source of protein, essential amino acids, oil, and metabolizable energy (Supplementary Table 1). Oil and soybean meal are the two main economical components of soybean (Warrington et al. 2015). While approximately 60% of the value of soybean comes from its meal, the remaining 40% comes from its oil (Pettersson and Pontoppidan 2013), therefore, the total content of protein and oil in soybean seed is more important than just its protein or oil content. A minimum of 47.5% protein in the soybean meal (with 12% moisture content) is required by marketplace and is needed for proper development of poultry and livestock fed with the soybean meal (Willis 2003). The meal protein value of recently released commodity soybean cultivars in the Midwestern US is generally below this minimum (http://unitedsoybean.org/). Such cultivars are inherently less valuable than their higher protein counterparts. This is a concern for the US soybean growers and breeders. Commercial soybean cultivars typically contain about 38–42% seed protein on a dry weight basis. The seed protein content needs to be at least 41.5% on a dry weight basis to produce meal with ≥47.5% protein from a soybean cultivar with 22% oil content (Willis 2003). If the seed oil content is lower than 22%, the seed protein content needs to be higher. A general rule of thumb is that protein plus oil in the seed should be ≥62.5% on dry weight basis to produce ≥47.5% meal protein (Hurburgh et al. 1990).

The poultry and swine industries have continued to improve their products to meet the demand of end users. For the past several decades, soybean meal has been the leading protein feed source for the animal and poultry production operations because of its high concentration of protein. Poultry and livestock industries use about 68 and 77% of the soybean meal consumed in the European Union and United States, respectively (http://www.soystats.com; http://www.fediol.be/). In addition, demand for soybean food products (e.g., whole beans, soymilk, tofu, bean sprouts, edamame) has increased fivefold (from $1 billion in year 2000 to $4.5 billion in 2013) due to increasing awareness of nutritional value and health benefits of soybean in the human diet (http://www.soyfoods.org/). Corn (*Z. mays*), sorghum (*S. bicolor*), and other cereal grains are also commonly fed to swine and poultry. Yet, these meals are commonly lower in protein content, crude fiber, and lower in tryptophan, threonine, isoleucine, and valine than soybean meal. Soybean meal is not an ideal protein source because it is typically (1) lower in the amino acids, methionine, and cysteine, often leading to additional supplements to the meal; (2) high in oligosaccharides that reduce metabolizable energy, and (3) high in potassium leading to higher moisture content in litter, which indirectly affects growth in chickens (*G. gallus domesticus*) (Youssef et al. 2011). In addition, some soybean proteins are antigenic (some individuals that eat soybean develop antibodies) and may cause allergies in a small portion of the population (He et al. 2015; Watanabe et al. 2016). The development of soybean cultivars with enhanced protein and amino acid content would further increase the economic value of the crop and will help to enrich the entire value chain from farmers to processors to end users.

**Fig. 1:**
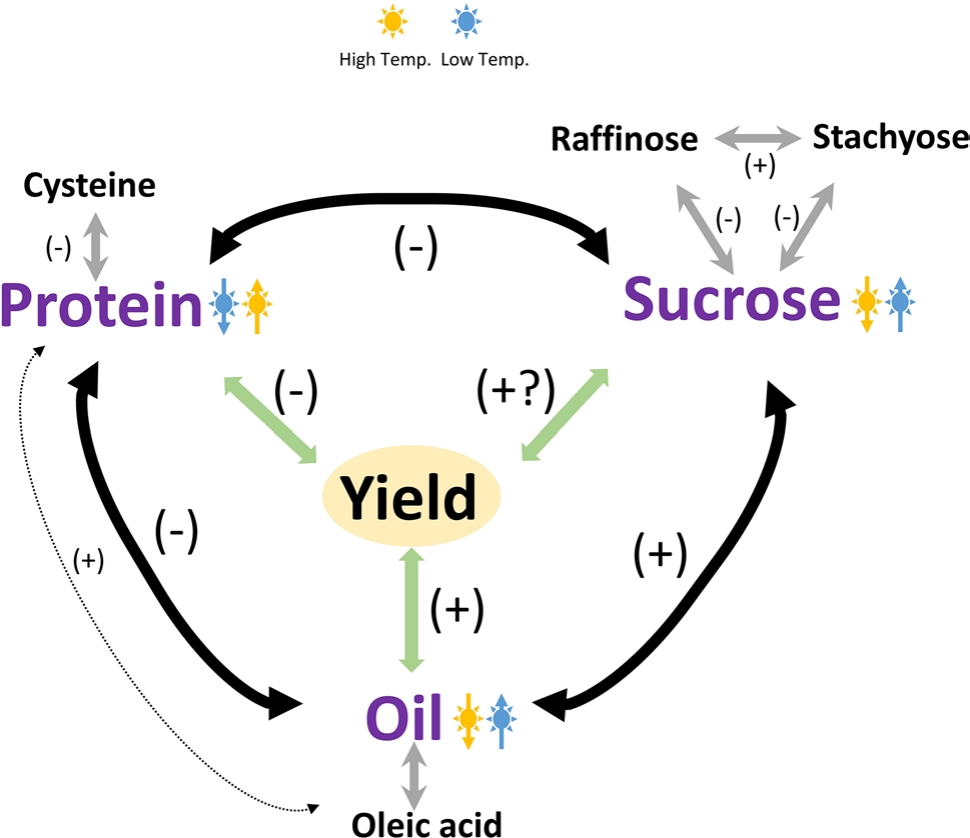
Correlations among different seed components and yield in soybean. Positive correlation (+), negative correlation (−). Temperature (*yellow* high temp; *blue* low temp.) affects protein, oil, and sucrose concentrations and it is shown by *up* (increase) and *down* (decrease) *arrows*. Correlation between known sub-components such as cysteine, oleic acid, raffinose, and stachyose are shown (color figure online)

**Fig. 2:**
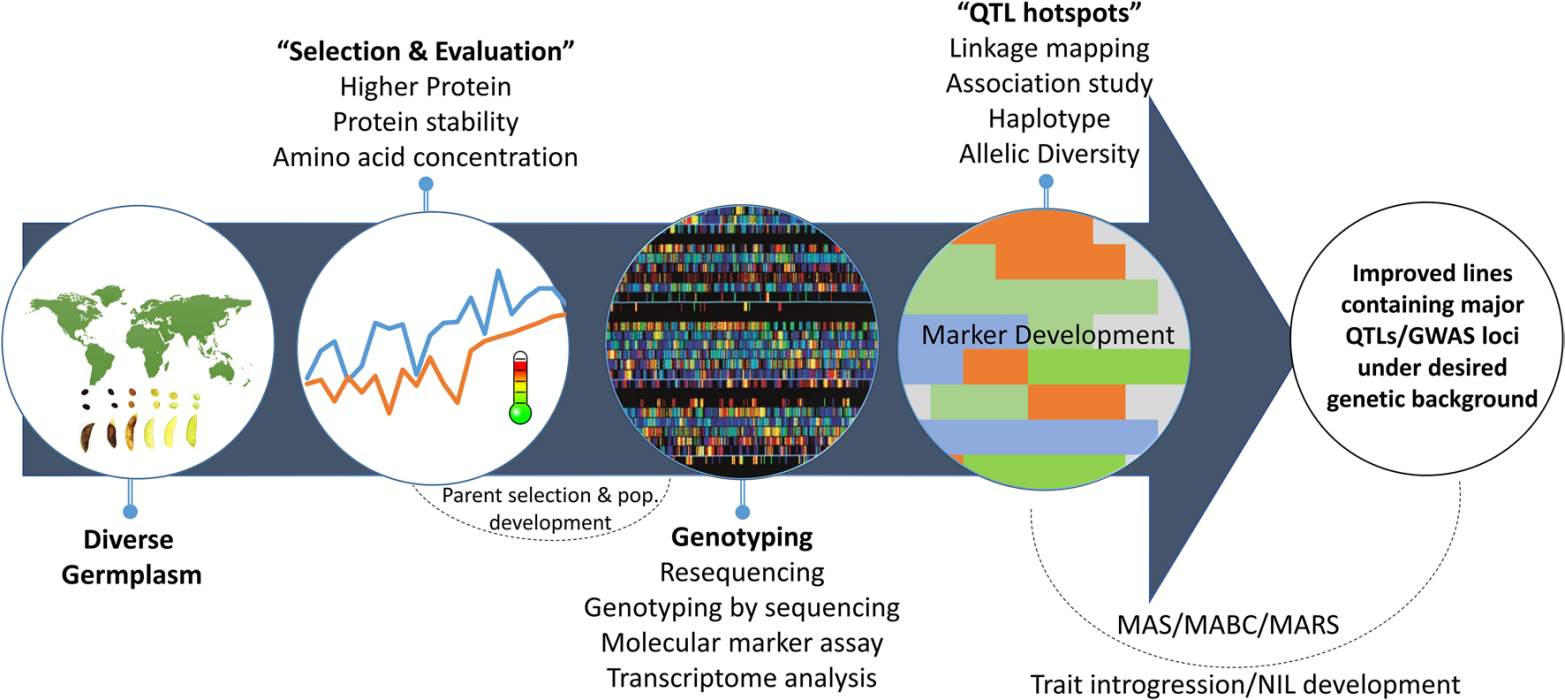
Integrated genomics and breeding approaches for soybean meal improvement. This figure illustrates the systematic approach for germplasm selection, phenotyping for seed components under different environments, parent selection, and development of mapping population. Different genotyping approaches can be used to identify the QTL hotspots, haplotypes, and markers. After validation, these QTL can be subsequently tracked in marker-assisted backcrossing (MABC) and marker-assisted recurrent selection (MARS) approaches for trait introgression and cultivar development. Additionally, available genomic resources and phenotypic data (training population) can be utilized in genomic selection of progeny and crossing design

Although steady genetic gains have been made in soybean yield through improved breeding strategies, the molecular and physiological mechanisms controlling yield, seed protein, and oil content are largely unknown. An understanding of the genetic and molecular control of soybean seed protein and oil could help identify strategies for developing better beans. Soybean seed composition, especially seed storage protein, is a complex trait that is controlled by multiple genes and affected by the environment and genotype × environment interaction (Carver et al. 1986; Chaudhary et al. 2015; Manjarrez-Sandoval et al. 1997). Increasing seed storage protein is difficult due to its strong negative correlation with oil content (Chaudhary et al. 2015) and seed yield (Bandillo et al. 2015; Chung et al. 2003; Kim et al. 2016). Figure 1 illustrates the overall correlation of different seed components in soybean. Genotyping with molecular markers is a common requirement for quantitative trait loci (QTL) mapping and genome-wide association studies (GWAS). Integration of these approaches aims to increase detection power of genomic loci (Fig. 2). Figure 2 illustrates investigation and integration of diverse germplasm followed by phenotyping and genotyping for identification of QTL hot spots for desired traits, which can be utilized in development of new germplasm. In this context, we reviewed the progress in genetic and genomics related to improvement of protein in soybean meal and highlighted the control factors that determine the protein content in the soybean meal.

## Global and economical perspective of soybean meal protein

### History, domestication, and utilization of soybean may relate to its protein content

**Fig. 3:**
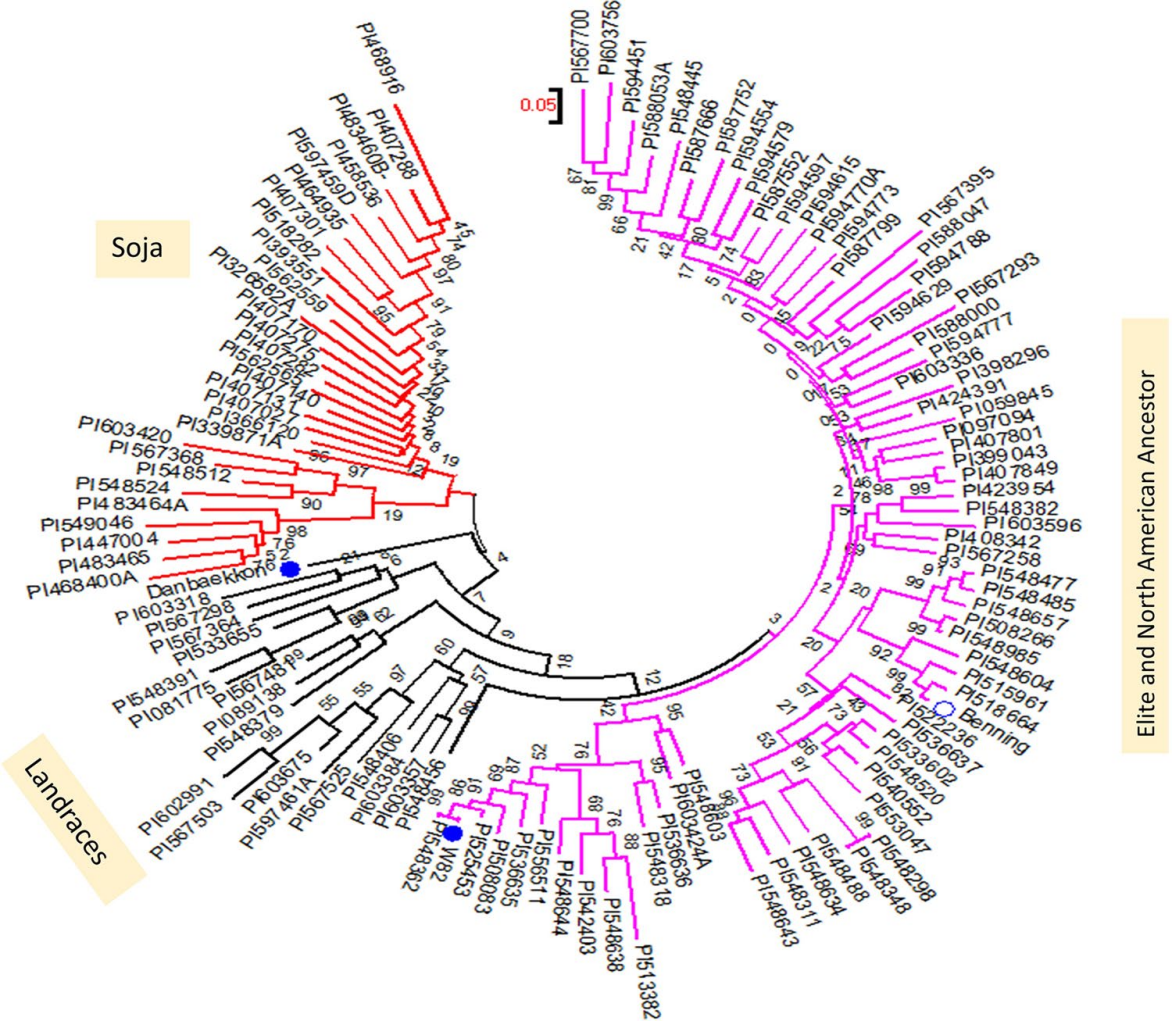
Analysis of the SNPs in the genomic regions (29.8–31.6 Mb) on Chr.20 shows that the North American ancestors and elite cultivars (*magenta*) are different as compared to soja (*red*) and several Korean cultivars/landraces (*black*). The known US (Williams82 and Benning) and Korean (Danbaekkong) elite lines are denoted by *blue dots* (color figure online)

Soybean originated in Southeast Asia and was first domesticated in China, then spread to Japan and Korea. North (http://ncsoy.org/) and South America (http://www.soyinfocenter.com/) did not begin cultivation of soybean until 1765 and 1882, respectively (Flaskrud 2003; Gibson and Benson 2005; Hymowitz and Shurtleff 2005). The Japanese and Korean accessions are closely related but generally distinct from Chinese accessions, and distinct from other parts of the world. The foundation of Northern American soybean breeding is derived mostly from two Chinese subpopulations, which reflects the composition of the American accessions (~90% are admixed) as a whole (Bandillo et al. 2015). Additionally, the American soybean germplasm resulted from a severe population bottleneck when they were introduced to North America (Hyten et al. 2006). About 17 North American Ancestor lines contributed 86% of the parentage of modern US cultivars. Similarly, Gizlice et al. (1993) reported that fewer than 15 progenitors constitute the major portion (>70%) of the genetic base for US soybean production. It has been reported that in addition to geographic origin, maturity groups were the principal determinants of population structure as well as seed composition traits within the soybean germplasm collection across the sub-continents (Fig. 3) (Bandillo et al. 2015).

Soybean breeding in South America is recent with a narrow germplasm base that is mainly derived from US cultivars and germplasm (dos Santos et al. 2016). The US has the longest history of soybean cultivation and utilization among countries in North and South America. Early use of soybeans in the USA was for forage and to some extent, green manure. Soybean production as a seed crop initiated in the 1940s (after World War II) as the demand for oil and lubricant increased (Gibson and Benson 2005). A commercial advantage due to higher yield and seed composition encouraged soybean breeders/farmers to develop improved cultivars. During 1950–1960s, demand for meat consumption increased, and meat producers found that soybean meal was the preferred source of protein for poultry and livestock at an affordable cost (http://ncsoy.org/). This use increased the demand for higher protein content and convinced breeders towards breeding for higher seed protein in recent years. On the other hand, soy foods including tofu, soymilk, natto, and edamame have generated tremendous interest especially with Korean and Japanese soybean breeders because of their nutritional value and human health benefits (Lee et al. 2015a; Shi et al. 2010). A history of the Korean breeding program and pedigree of popular lines suggest that soybean breeding focused on soy-food-driven traits especially protein and other functional ingredients (Lee et al. 2015a).

### Major soybean meal producers: US, Brazil, and Argentina

Despite the domestication of soybean in Asia, it found a welcome home in US and became successful as forage crop and subsequently a major grain crop because of the need for oil (food) and meal (feed), and it benefited other crops in rotation (Brown 2012). The success of soybean in US created research interest in Brazil for developing soybean that could be grown at lower latitudes (Goldsmith and Hirsch 2006). During the past ~80 years of soybean breeding in the US and ~40 years in Brazil, the increase in soybean production has been significant, and was built upon a consistent effort in germplasm exchange, advanced breeding techniques, and agronomic practices. On the other hand, Southeast Asian countries (China, Japan, and Korea) have fewer hectares devoted to production because of the historical dominance of US soybean production, and political decisions to focus on Southeast Asia being self-sufficient in cereal grains (rice and wheat) (Brown 2012).

**Fig. 4:**
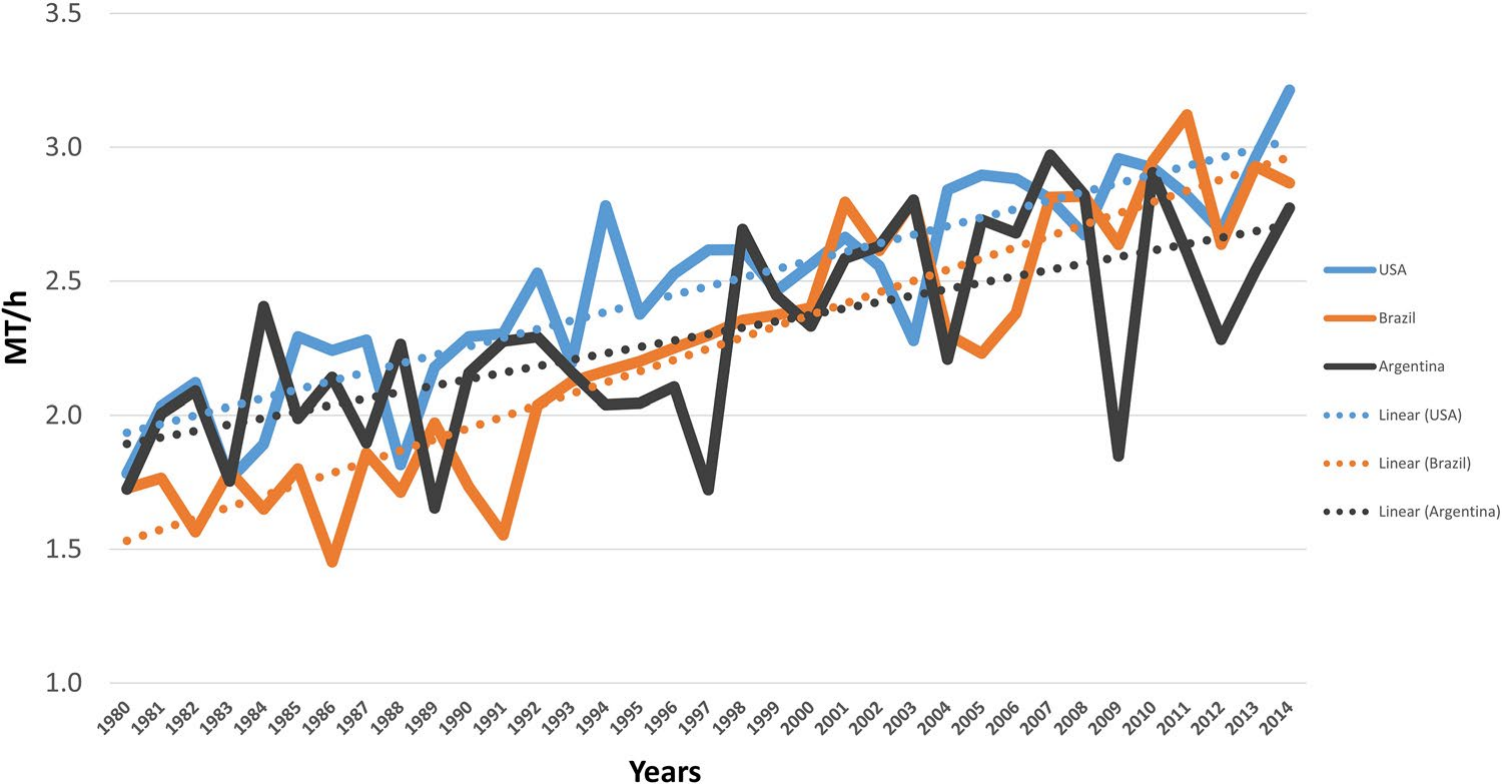
Soybean production and growth trend in USA, Brazil, and Argentina from 1980 to 2014

The United States leads the world in soybean production with 34% [108 Million Metric Tons (MMT)] followed by Brazil with 30% (94.5 MMT), and Argentina with 18% (56 MMT) (SoyStats 2014, http://www.soystats.com). In the US, a larger portion of soybean (79%) is intended for export and a small amount is used for human food (~2%), and the rest (~18%) are processed to extract seed oil, and the resulting protein meal undergoes complex processing to make defatted soybean meal (http://www.soymeal.org/). In 2015, about 31% of the crop area (82.7 M acres) was planted to soybean in the US, which was slightly less than corn planting (34%). There was an increase of 6.9 M acres in soybean planting compared to that of 2013. Soybean production in Brazil and Argentina has grown rapidly in the recent years and soybean exports have grown accordingly. The average soybean yield (from 1980 to 2014) in the US (2.5 MT/h) exceeded that of Brazil (2.2 MT/h) and Argentina (2.3 MT/h) (Fig. 4). Importantly, Brazilian soybean production has shown a rapid and significant increase overtaking Argentina during early the 2000s and approaching total US soybean production (Fig. 4). Major soybean producing countries other than those in North and South America, include China and India which produce about 12.4 and 10.5 MMT soybean, respectively.

The US dominates world soybean production and export, however, Argentina (45%) leads the world soybean meal export followed by Brazil (22%) and US (18%). This is because about 75% of soybean meal produced in the US is used domestically (USDA; World Agri. Supply and Demand Estimates (WASDE), Feb. 2016). Growing conditions as well as processing techniques affect the digestibility of amino acids in the soybean meal. To determine the relative feeding value of alternative protein sources, the lysine level is estimated, which in-turn determines the comparative economic value of the protein source. A comparative study of soybean meal from Argentina, Brazil and the US showed that the US soybean meal samples contain relatively higher lysine, cysteine, and threonine levels compared with South American soybean meal. Similarly, Ravindran et al. (2014) and Kim et al. (2008) conducted a survey of soybean meals from different origins to study the nutrient profile, metabolizable energy, and digestible amino acids of broiler chickens (Kim et al. 2008; Ravindran et al. 2014). In that study, samples from US exceeded other countries in crude protein, apparent metabolizable energy (AME), lysine, and methionine concentrations (Ravindran et al. 2014). This is an important finding that emphasizes the value of amino acids in US soybeans, which have been previously thought to be inferior to those from South America.

## Genetic analyses of protein content

Soybean seed protein is a quantitatively inherited trait and controlled by many genes and regulators. With the advancement of genetic map construction (Hyten et al. 2010; Lee et al. 2015b), the availability of a well-annotated reference genome (Schmutz et al. 2010), resources for association mapping (Song et al. 2013, 2015), and whole-genome resequencing (WGRS) data (Valliyodan et al. 2016; Zhou et al. 2015), a large number of QTL for seed protein content have been identified (Supplementary Table 2). Over the past two decades, more than 160 QTL from 35 independent studies have been reported for seed protein content in soybean (Supplementary Table 2). Among these, a major QTL for seed protein and oil content (Diers et al. 1992; Hwang et al. 2014; Qi et al. 2016; Warrington et al. 2015) has been consistently mapped on Chr. 20 and remarkable attention has been given to this QTL because of its high additive effect and stability (Lestari et al. 2013). However, due to the lack of large effect and stability, negative relationship with oil/yield, and inconsistency across environments, very few protein QTL were further used or incorporated in breeding programs (Wang et al. 2015). Unstable QTL can give unreliable data when applied in marker-assisted selection (MAS) scheme and, therefore, a stable and consistent QTL with large effect is desirable for use in soybean breeding (Kadam et al. 2015; Panthee et al. 2005). In addition to these genetic-environmental factors, lack of economic incentive for higher protein cultivars is one of the primary impediment for the development of such cultivars. According to the Soybean Genetics Committee (http://www.soybase.org), only two QTL, one on Chr. 15 (cqPro-15) and another one on Chr. 20 (cqPro-20) are designated as officially confirmed QTL based on error rate (lower than 0.01) and confirmation study showing alleles at the same locus are segregating in all the test populations (http://soybase.org/). The QTL on Chr. 20 has been the focus of several studies, including the testing of the high protein allele in different genetic backgrounds and showed a large additive effect (Warrington et al. 2015). Recently, Kim et al. (2016) identified QTL for higher protein and lower oil content from PI 407788A that mapped to Chr. 15. In another study, Warrington et al. (2015) identified and mapped a major QTL for seed protein and amino acid content on Chr. 20 in the Benning × Danbaekkong population and showed that a favorable allele from Danbaekkong imparts a total of 55% of the phenotypic variation in seed protein content. The favorable allele exerted little negative drag on seed yield in that population. However, in other studies using different high-protein sources, the presence of QTL for higher protein on Chr. 20 was negatively correlated with seed yield (Chung et al. 2003; Nichols et al. 2006; Sebolt et al. 2000). This suggests that Danbaekkong may have a different allele than these sources or may have a genetic background that mitigates the yield drag of the QTL. Another source, BARC-7 (Leffel 1992), was successfully utilized by Chen et al. (2008, 2011) to develop high-protein soybean germplasm and commercial varieties. Subsequent genetic analysis identified the same major QTL on Chr. 20 and a new QTL on Chr. 14, suggesting BARC-7 may carry alleles different from Danbaekkong.

To better understand the genomic loci regulating seed protein content, we summarized the QTL information from previous bi-parental mapping populations, populations genotyped by WGRS and SoySNP50K BeadChips (Table 1). The majority of these QTLs are on Chrs. 20, 15, 18, and 6 (Supplementary Table 2). QTL including the major one on Chr. 20 were frequently detected in a similar genomic region based on GWAS of diverse germplasm populations and linkage association analysis (Bandillo et al. 2015; Hwang et al. 2014; Sonah et al. 2015; Vaughn et al. 2014). However, the significant level (−log10 values) of the QTL on Chr. 20 based on GWAS varied among reports. The difference could be explained by source of population, population size, recombination rate, or extent of linkage disequilibrium (LD) associated with the germplasm panels. The LD reflects dependence of alleles at different loci and is central to both QTL detection and MAS (Dekkers and Van der Werf 2007). Bandillo et al. (2015) performed an association mapping study using the publically available SoySNP50K data (Song et al. 2015) in over 12,000 *G. max* accessions and identified GWAS signals on Chr. 15 and 20 for protein concentration. They determined that the GWAS signals on these two chromosomes were more propounding for Korean accessions, and the frequency of these high protein alleles was lower in Chinese and US accessions. Genome-wide association study provides greater QTL detection power and map resolution but it also limits detection of rare variants that are usually filtered from a subset of the large population (Phansak et al. 2016). In a recent study, Phansak et al. (2016) performed selective genotyping of multiple bi-parental populations to mitigate the GWAS rare-variant problem, and identified significant QTL for protein and oil in 48 donor lines. That study confirmed that rare and common QTL can be detected using a selective genotyping strategy.

**Table 1:** Major seed protein QTL identified using bi-parental mapping population and diverse germplasm (GWAS)

P1	P2	Major QTL (Chr.)	Additive parent	References
QTL linkage mapping				
A81356022	PI 468916	20, 15, 18	PI 468916	Diers et al. (1992)
		20	PI 468916	Nichols et al. (2006)
M82806	HHP	20, 15, 18	HHP	Brummer et al. (1997)
Young	PI 416937	15	Young	Lee et al. (1996)
Parker	PI 468916	20	*G. soja* (PI 468916)	Sebolt et al. (2000)
A3733	PI 437088A	20	PI 437088A	Chung et al. (2003)
PI 97100	Coker 237	20, 15		Fasoula et al. (2004)
Essex	Williams	6	Essex	Hyten et al. (2004)
N87-984-16	TN93-99	18	TN93-99	Panthee et al. (2005)
ZDD09454	Yudou12	20, 18		Lu et al. (2013)
Magellan	PI 438489B	15, 5, 6	Magellan	Pathan et al. (2013)
Magellan	PI 567516C		Magellan	
R05-1415	R05-638	14, 20	R05-1415	Wang et al. (2015)
Benning	Danbaekkong	20, 15	Danbaekkong	Warrington et al. (2015)
William 82	PI 483460B	6, 20, 15	PI 483460B	Patil et al. unpublished
Multi-population (48 F_2_ pop.)		20, 15, 10		Phansak et al. (2016)

Pop size	Markers	Major loci (Chr.)	References
298 accessions	SoySNP50K	20	Hwang et al. (2014)
3K accessions	SoySNP50K	20	Vaughn et al. (2014)
139 accessions	GBS-47K	20, 5, 8	Sonah et al. (2015)
302 accessions	WGRS	13, 03, 17, 12, 11, 15	Zhou et al. (2015)
>12K accessions	SoySNP50K	20, 15, 6	Bandillo et al. (2015)
106 accessions	WGRS	20	Valliyodan et al. (2016)

The PI with underline denotes wild soybean (*G. soja*)

Consistent with the reports of Vaughn et al. (2014) and Bandillo et al. (2015), we observed that the *G. max* accessions from Korea contains a relatively higher amount of seed protein than those from the US and other countries. This possibly have resulted from breeding strategies for higher protein content, attributed to a breeding focus on soy-protein food products. To understand the genomic variation, we performed a genome-wide phylogenetic analysis for a Korean soybean cultivar, Danbaekkong, elite North American ancestors (NAA), Asian landraces, and several wild soybean lines (*G. soja*) using the SoySNP50K data set. The analysis result in three clades where Danbaekkong clustered with NAA as expected (data not shown). However, when the SNPs in the 27–32 Mb on Chr. 20 were analyzed, the Danbaekkong clustered separately from NAA as well as landraces and *G. soja* accessions (Fig. 3). This observation indicated that most of the commercial soybean cultivars in the US are fixed for the low protein allele at major QTL on Chr. 20, suggesting that introgression of the desired high protein allele from Danbaekkong into an existing US elite soybean background would enhance seed protein content.

It is known that polyploidization is a crucial force in plant evolution and domestication. Genome duplication events leading to polyploidization in soybean appeared approximately 59 and 13 million years ago (MYA) and about 75% of the genes in soybeans are present in multiple copies (Schmutz et al. 2010). The duplicated genomes/genes could be important for acquiring differential functionality through neo-functionalization or sub-functionalization (Patil et al. 2015). However, the duplicated genome increases the epigenetic complexity, results in epistatic interaction (non-additive) gene regulation, and limits the detection power of true QTL (Comai 2005). Growing evidence from synteny studies showed that duplicated QTL on another chromosome leads to underestimation of the effect of real QTL (Kearsey and Farquhar 1998; Lestari et al. 2013). To understand genome complexity in terms of QTL synteny, we analyzed two major protein QTL (Chr. 20 and 15) and segmental duplication blocks between the regions. In agreement with Lestari et al. (2013) we identified the syntenic block of Chr. 20 QTL on Chr. 10 (Supplementary Figure 1). Although Lestari et al. (2013) reported the QTL was between 24.8 and 28.8 Mbp, the QTL was most likely in the genomic region of 29.8–31.6 Mbp which was supported by integrating GWAS, transcriptome, and QTL mapping analysis (Table 1) (Bandillo et al. 2015). In addition, we analyzed conserved gene order and identified 18 genes that were tandemly duplicated on Chr. 10 and showed similar gene ontology (Supplementary Figure 1; Supplementary Table 4). It is noteworthy that the three putative candidate genes identified by Bandillo et al. (2015) are present only on Chr. 20 and suggest that these non-duplicated genes might be related to protein content. Similarly, Chr. 15 QTL (38.1–39.7 Mbp) showed an inversely duplicated genomic block on Chr. 8 (Supplementary Table 4). The QTL on Chr. 15 comprises 18 putative genes, 13 of which were duplicated with similar gene function. This syntenic analysis provided a basis for divergence of QTL regions that took place during recent genome duplication and suggested the retention or loss of several genes that might be responsible for protein and oil content in soybean.

In addition to conventional breeding, transgenic approaches have also been utilized for protein and amino acid improvement and functional studies. Schmidt et al. (2011) suppressed glycinin and conglycinin seed storage protein in soybean and observed that mutant lines maintained the level of protein and oil, similar to non-transgenic lines suggesting proteome rebalancing in seed. Similarly, the level of allergens were reduced by suppressing the β-subunit of 7S globulin using RNAi technology (Qu et al. 2015). In another study, a successful attempt was made to partition the carbon and nitrogen for protein improvement independent of the genetic background and original cultivar protein content (Li et al. 2015). A unique qua-quine starch (QQS) gene from Arabidopsis, regulates metabolic processes affecting carbon and nitrogen partitioning among protein and carbohydrates. This gene was expressed in soybean seed and increased seed protein (8–10%) by decreasing starch levels. The expression of the gene did not affect the plant morphological traits (plant height, seed size, color, etc.) as well as yield and oil content (Li et al. 2015). Recently, a multi-gene stack approach was used to develop what could potentially make a sustainable soybean-based feedstock for aquaculture. (Park et al. 2016). In this study, they improved EPA (eicosapentaenoic acid) and astaxanthin concentrations resulting in a novel oil trait without affecting protein quality.

## Genetic analyses for amino acid composition

**Fig. 5:**
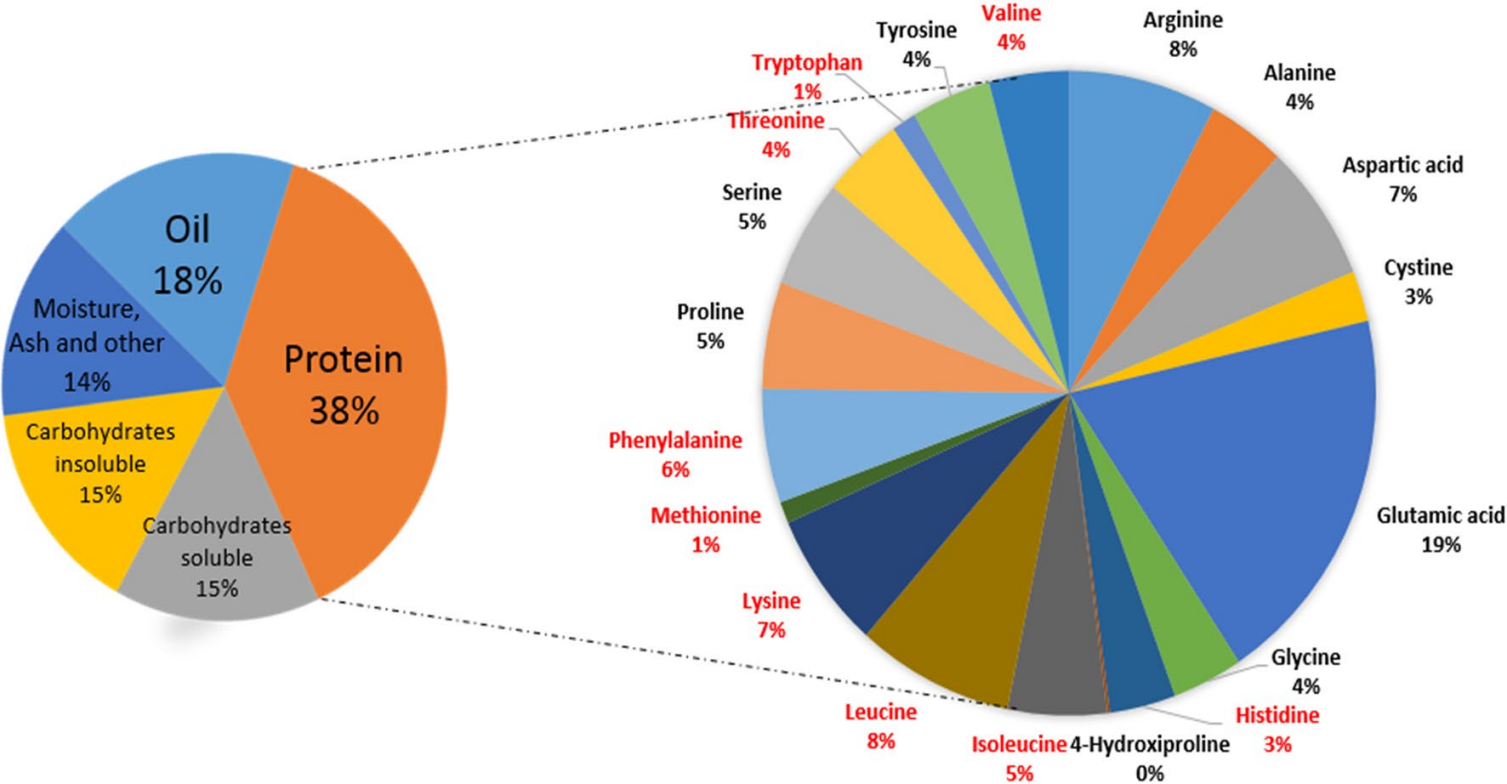
Amino acid composition of soybean protein. *Red* essential amino acid (EAA), *black* nonessential AA. Data source: Asif and Acharya (2013), Berk et al. (1992), Kuiken et al. (1949) (color figure online)

The major function of protein meal in nutrition is to supply sufficient amounts of essential amino acids. Based on solubility properties, globulins and albumins are two major components of dicot seed storage protein, and soybean primarily belongs to the globulin (~70%) family (Mandal and Mandal 2000). The soybean globulins (glycinin and β-conglycinin) are relatively low in sulfur-containing amino acids methionine (Met) and cysteine (Cys) as well as threonine (Thr) and lysine (Lys) (Warrington et al. 2015). Increasing the soybean storage protein content of seed while improving the ratio of glycinin to β-conglycinin is of great potential importance for soybean improvement (Ma et al. 2016; Panthee et al. 2006). Importantly, monogastric animals cannot synthesize these essential amino acids and, therefore, need to obtain them from their diet (Fig. 5). Due to these deficiencies, producers of poultry and swine spend substantial amounts for additional synthetic supplements and distiller’s dried grains with soluble DDGS from the marketplace. Therefore, besides increased protein content, enhancing sulfur-containing amino acids (Met, Thr, Cys, and Lys) would improve the nutritional value of soybean meal. More than 70% of the essential amino acid-enriched meal is used in the feed industry (Chaudhary et al. 2015; Warrington et al. 2015).

Although soybean cultivars with improved protein content have been successfully developed, only a few studies have been conducted to evaluate amino acid content or to identify genomic regions controlling amino acid composition. The difficulty in breeding for improved amino acids could be due to lack of genetic variability, lack of high-throughput, and cost-effective phenotyping platform to screen a large number of samples for amino acids. A survey of the public database (SoyBase) revealed limited studies of QTL for amino acid composition (Supplementary Table 3). Panthee et al. (2006) identified QTL for essential amino acids in a F_6_-derived recombinant inbred population (Supplementary Table 3). In a recent study, major QTL for essential amino acids and crude protein were identified on Chr. 20 in the Benning × Danbaekkong population (Warrington et al. 2015). It was also noted that a favorable allele for amino acids was inherited from cv. Benning, while an allele for improved seed protein content and a reduced level of these amino acids was contributed by cv. Danbaekkong. Moreover, negative correlations of crude protein with Lys and Thr and a positive correlation between Thr with Lys were also observed (Warrington et al. 2015). Among the essential amino acids, Met, Lys, and Thr are synthesized from a common precursor aspartate, thus, they are strongly correlated. In another study, Krishnan et al. (2015) introgressed leginsulin (Cys rich protein) and a high-protein trait from an Asian soybean germplasm, PI 427138, into North American experimental line (LD00-3309). While they were successful in introgressing leginsulin and improving protein content, the overall concentration of sulfur-containing amino acids was not changed compared to parental lines.

In addition to mapping studies, transgenic technologies have been utilized in attempt to improve essential amino acids in soybean and also other crop plants (Altenbach et al. 1989; Falco et al. 1995; Kim and Krishnan 2004; Kortt et al. 1991). The overexpression of methionine-rich proteins resulted in little or no improvement in amino acid concentrations. In another study, the bacterial dihydrodipicolinate synthase (DHDPS) gene and aspartokinase (AK) were expressed in seed-specific tissue in canola (*B. napus*) and soybean, which significantly improved the lysine content by twofold, however, the germination and seed quality were poor (Falco et al. 1995). Recently, Yang et al. (2016) conducted a proteomic study to investigate and identify differences in the proteome of high and low protein soybean seed as well as with and without globulin subunit 11SA4. They demonstrated the interaction of protein content and absence of 11SA4 subunit that modulates the protein profile, and improvement in gelling properties of tofu and globulin composition (Yang et al. 2016). In another study, soybean embryonic cultures transformed with anthranilate synthase gene (first enzyme in Trp biosynthesis) showed increased free Trp levels in seed and leaves; yet a marginal increase was observed in total seed Trp (Inaba et al. 2007). These results indicate that the amino acid levels are not only dependent on their transport, but also on their de novo synthesis and sequestration into the transgene product.

## Rate-limiting factors of protein improvement

### Temperature effects and environmental stability (ES)

**Fig. 6:**
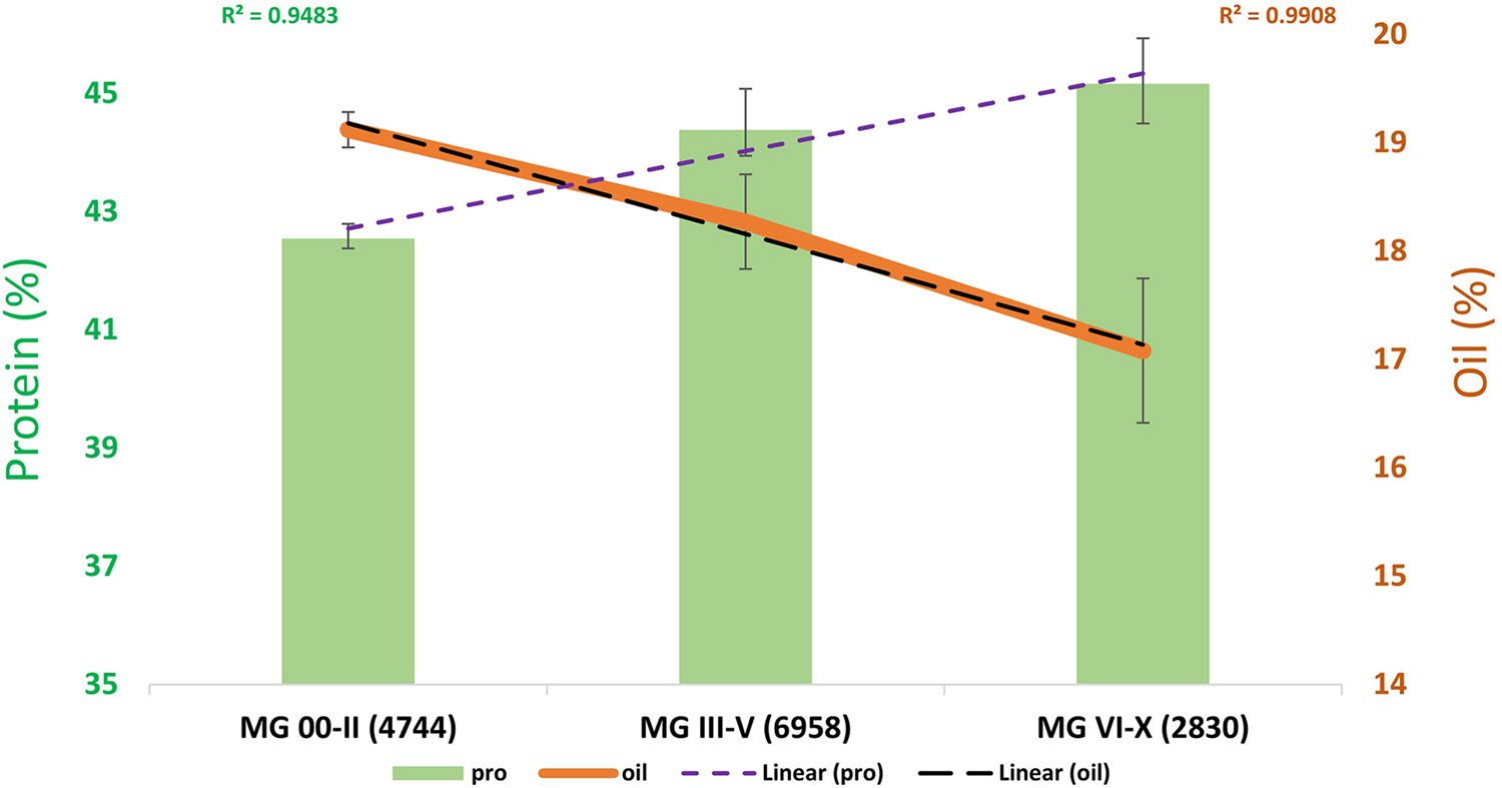
Correlation of maturity (temperature effect) with seed protein and oil content in *G. max* accession. Number of *lines* in *bracket*

To understand the global overview of protein variation in different maturity groups, we utilized the GRIN (http://www.ars-grin.gov/) phenotypic data for total seed protein content. The phenotypic variation showed the advantage of protein content in late maturity group (MG V—X) lines compared to early maturity group lines (MG 000—II) (Fig. 6). It has been estimated that the genetic variation explained by geographic regions (~5%) is higher than that explained by the maturity group (~2%) (Gizlice et al. 1993; Bandillo et al. 2015), suggesting that genomic difference due to geographical origins impart more variation in seed composition phenotypes (protein and others). Soybean breeders in the Northern states may attempt to overcome the effect of lower temperature on seed composition traits by selecting more diverse parental lines in early maturity groups to broaden genetic potential (Piper and Boote 1999). In two other studies, the temperature effect on oil and protein content in ten maturity groups was evaluated (Piper and Boote 1999; Thomas et al. 2003). They found that oil content increased and approached a maximum at 28 °C, however, above 28 °C the oil content declined significantly. Importantly, they also identified that transcript abundance for non-structural carbohydrates, auxin-downregulated gene (ADR12), and β-glucosidase was decreased significantly at elevated temperatures (Thomas et al. 2003). Wolf et al. (1982) studied the effect of temperature under controlled greenhouse conditions (five temperature regimes) on soybean seed constituents (oil, protein, amino acids, and fatty acids). They found that protein content was positively correlated with higher temperature and the amino acid concentration (except methionine) remained unchanged (Wolf et al. 1982). These analyses helped to explain whether genetic or temperature factors cause regional difference in protein content in soybean seed. Dornbos and Mullen (1992) observed that seeds from plants exposed to a temperature of 35 °C during seed filling contained ~4% more protein than plants exposed to 29 °C. In other studies, researchers observed that transferring soybean plants to a higher temperature at reproductive stages (R1–R7) affected plant vigor and overall yield (Egli et al. 2005; Keigley and Mullen 1986; Ren et al. 2009). Additionally, proteomic studies showed that seed protein expression profiles changed significantly under high temperature (Ren et al. 2009). The key enzyme in the methionine biosynthetic pathway was upregulated during the early stage of high-temperature treatment; however, the molecular basis of interaction between temperature and seed components are still largely unknown (Ren et al. 2009).

It is a well-known phenomenon that day length (photoperiod) and day/night temperature (circadian cycle) controls flowering time (Kinmonth-Schultz et al. 2016), subsequently regulating post-flowering and vegetative growth via energy source transport and allocation (especially carbon) to the developing seeds (Patil 2016; Xu et al. 2013). It has been reported that N and P concentrations in mature seeds increase with increase in day/night temperatures (Thomas 2001). This observation leads to a speculation that there might be some genes/loci (N transporter genes, e.g., NRTs) that show optimum activity of N uptake from soil and transport to the seeds at elevated/optimum temperature conditions. Very little information is available about circadian rhythms in developing seeds, which could be important for determining the extent and timing of resource allocation in developing seeds (Hudson 2010; Patil et al. 2015; Syed et al. 2015). It has been speculated that post-flowering reproductive development is regulated by phyA genes—*E3* and *E4*. Hudson (2010) performed transcriptome profiling of developing soybean seeds under day-/night-controlled condition and identified a subset of circadian-regulated genes related to carbon metabolism. They also showed that genes involved in protein biosynthesis are regulated (*cis*-regulation) by circadian cycle.

An important goal of soybean breeders is to achieve higher yield without losing protein and oil. Therefore, additional research should emphasize screening, identification, and understanding the genetic regulation of protein stability under different temperature regimes without affecting yield and oil content. Combining genetic studies, expression profiling and exploring natural genetic variants may advance our understanding of temperature stable protein.

### Genetic architecture, negative correlation with oil and yield

#### Background and allelic effect

Seed protein content and composition are dependent on several factors. The genetic background of an elite parent plays an important role in the phenotypic expression of a newly introgressed alleles because of complex epistatic interactions (Deshmukh et al. 2014). Most of the QTL affecting seed protein and yield and yield-related components were detectable only in one of the parental genetic background (GB) in introgression lines of reciprocal crosses (Yates 2006). Yates (2006) identified that the high protein allele within a different genetic background resulted into reduced Thr and Lys content. The high protein allele from Danbaekkong on Chr. 20 has been demonstrated to increase seed protein content in several maturity groups (III–VIII) in various genetic backgrounds with little drag on seed yield (Mian et al. 2017). On the other hand, the protein QTL alleles on Chr. 20 from other sources, including wild *G. soja* showed yield drag (Chung et al. 2003; Nichols et al. 2006). Based on the aforementioned allelic arrays and their effects on crude protein and the amino acid profile, it is not feasible to select only for the major crude protein QTL on Chr. 20 and improve protein quality. By selecting for the Danbaekkong allele on Chr. 20 and for either Danbaekkong or Benning alleles at QTL on other chromosomes, which affect protein quality, breeders may be able to improve protein and maintain protein quality concurrently.

In plants, there is considerable evidence demonstrating that the expression of genes and/or QTL for agronomic traits are strongly affected by the genetic background (Patil et al. 2016) and furthermore this effect strongly influences application of QTL to MAS and breeding practice. Although a large number of markers are available (Patil et al. 2017), MAS for protein improvement has not been widely utilized by breeders and this may be due in part to the complexity of the soybean genome (tandem duplication) and extent of LD that they have in the population with loci that contribute to genetic variation. These issues prevent the selection of desired recombination events for the trait (Dekkers and Van der Werf 2007). In addition, genetic effects related to epistasis are either poorly estimated or ignored. Other inherent limitations to MAS are related to the estimates of QTL position, stability of QTL across environments, genetic effect, and the rates of false positive/negative loci (Ragot et al. 2007).

#### Pleiotropic effect

Previously, researchers have observed that protein and oil have negative correlation because of either tightly linked QTL or pleiotropic effects associated with competition for nutrient allocation (Diers et al. 1992; Nichols et al. 2006; Pathan et al. 2013; Sebolt et al. 2000). The balance between carbon (C) and nitrogen (N) supply impacts the final composition of developing seeds as well as flux transport. Protein content of soybean seed is significantly affected by the N uptake during early seed development (Krishnan 2001; Paek et al. 1997). In later stages of seed development, altered N supply (in form of glutamine) does not dramatically impact relative amino acid or storage protein content, suggesting that seed metabolism accommodates different levels of protein biosynthesis, however, it maintains a consistent rate of dry weight accumulation (Allen and Young 2013; Patil et al. 2009). Another apparent reason is that, seed component calculations are constrained to total 100%, therefore, increasing one would decrease other component mathematically. Pleiotropy is a ubiquitous phenomenon in organisms that has important influence on metabolic genetics and evolutionary biology (Stearns 2010). Several genome-wide association studies (Bandillo et al. 2015; Hwang et al. 2014; Vaughn et al. 2014) and QTL analysis (Nichols et al. 2006; Pathan et al. 2013) have shown similar QTL, haplotype or genomic loci (e.g. Chrs. 20, 15, and 5) for oil and protein indicating negative pleiotropic effect or linkage (larger LD). In addition to pleiotropic effects of protein on oil and yield, variation in seed protein concentration significantly affects seed size, crop growth, and development (Poeta et al. 2016). In that study the authors reported that high-protein genotypes showed lower leaf area and harvest index when compared with high-yielding genotypes. In addition, high-protein large seed was associated with more assimilate availability per seed during seed filling, while high-protein small seed showed higher leaf area at the beginning of seed fill, more canopy biomass production, and low levels of assimilate per seed (Poeta et al. 2016). These results supported the fact that high-protein genotypes impacts plant growth and development via assimilate transport. Therefore, breaking the undesirable genetic linkage between protein, oil, and yield-related loci through repetitive recombination and random mating is necessary.

### Points to consider for understanding and improving genetic basis of soybean meal protein

#### Genomics-assisted breeding (GAB)

The integration of genomic tools and breeding practices are the core components of genomics-assisted breeding for developing improved cultivars for any given trait. Figure 2 illustrates the major steps involved in developing an improved cultivar. Near isogenic lines (NILs) are important genetic resources that can be utilized for comparative physiological and biochemical studies to understand the function of a single gene or a major QTL, and they allow for differentiation of the effect of a gene/QTL from the background genome effect. NILs can be developed for major QTL (e.g., protein QTL on Chr. 20) by backcross breeding. Using NILs, the effect of a QTL and the phenotype it produces (i.e., protein or amino acid content) can be estimated precisely without the confounding effects of differences in genetic backgrounds. Additionally, environment and maturity affect seed protein content, therefore, developing NILs in a range of maturity groups is desirable. In a recent study, marker-assisted backcrossing selection approach was utilized to produce a NIL-(cgy-2–NIL) containing mutant cgy-2 allele, responsible for the absence of allergenic α-subunit of β-conglycinin (Song et al. 2014). It is also possible to incorporate multiple genes/QTL into elite lines in a cyclic forward crossing scheme using markers to track the target gene/QTLs. However, in majority of cases, introgressing desirable gene combinations or pyramiding several QTLs (as in the case of seed protein content) through marker-assisted backcrossing (MABC) can be challenging and hence, marker-assisted recurrent selection (MARS) should be employed as an effective approach (Brim and Burton 1979; Holbrook et al. 1989; Lewers and Palmer 1997; Varshney et al. 2013). Earlier, Brim and Burton (1979) successfully utilized four cycles of recurrent selection for increased gain yield, protein, oil, and oleic acid content. Furthermore, the next-generation sequencing (NGS) data can be used effectively for genomic selection (GS) to identify desirable parents and progenies for future germplasm development. Recently, Jarquin et al. (2016) assessed the genomic and phenotypic data of over 9000 accessions and developed genomic predication models to evaluate the genetic value for protein, oil, and yield traits. Their preliminary prediction models can be utilized further in selecting parents for cultivar development. Similarly, genomics-assisted haplotype analysis is a promising approach if the information of major QTL is available and that can be applied to select desirable haplotype blocks for parental selection and crossing-by-design (Patil et al. 2016).

#### Germplasm screening under cool and warm environment

As discussed above, the cooler temperature affects soybean seed protein content negatively, suggesting that ambient temperature during seed development has propounding effect on soybean seed protein (Figs. 1, 6). According to Piper and Boote (1999) the Southern US cultivars had a higher genetic potential for protein and, therefore, higher protein concentration can be explained by greater genetic potential as well as higher temperature during maturity. Hence, it is desirable to evaluate diverse soybean germplasm and adapted elite cultivars/lines under cooler and relatively warmer temperatures in the field to discern environmental effects on soybean protein. In addition, screening NILs with different protein QTL and maturity groups under different temperature regimes will help understand the QTL effect. Under the soybean meal improvement project sponsored by United Soybean Board (USB), an environmental stability study (ESS) is being initiated to discover new sources of high and stable protein. Followed by the initial ESS study, the stable lines need to be screened under controlled conditions for further confirmation. A dedicated study is required to investigate the effect of cool/warm temperature on global expression profiles to identify genes/TFs associated with protein stability.

#### Wild soybean, an important gene pool and “introgression library”

Large germplasm collections serve as an important source of genetic diversity and phenotypic variation for crop enhancement, especially if that crop has narrow genetic diversity. The wild species are considered reservoirs of novel alleles for traits that might have been selected out or ignored during domestication and breeding (as in the case of protein content). Researchers have successfully introgressed the high protein allele from cv. Danbaekkong into locally adapted lines, however, it may reduce the concentration of some essential amino acids (unpublished). Therefore, it may be necessary to utilize wild species accessions as introgression libraries as well as developing inter-specific populations. On the other hand, landraces and elite cultivars can be used to develop mapping populations, reference/core sets and training populations, and elite breeding lines can be included in training populations (Varshney et al. 2013). Superior alleles for some traits (e.g., disease resistance, abiotic stress tolerance and yield) have been identified and transferred from the wild species to elite cultivars (Delheimer 2012; Prince et al. 2015; Tanksley 2004). Wild soybean (*G. soja*) is a unique resource to study regulation of protein and amino acid biosynthesis because the seed concentration of these components are higher in *G. soja* compared with *G. max.* However, these resources are still mostly untapped by breeders and others in soybean research community. The reason for underutilization of wild soybean for seed composition could be due to linkage drag on favorable agronomic characteristics (Asekova et al. 2016; Sebolt et al. 2000). However, this issue could be resolved by advanced backcross QTL-based breeding, which was utilized for introgressing alleles from wild tomato for yield improvement (Tanksley and Nelson 1996).

#### Precise phenotyping for protein and amino acids (common phenotyping platforms, assay, and equations)

Advances in next-generation omic technologies (genomics, transcriptomics, and proteomics) have benefited some breeders and accelerated the potential rate of genetic improvement by molecular breeding (Chaudhary et al. 2015; Chen et al. 2016). In general, the lack of precise, high-throughput and cost-effective phenotyping capabilities limits our ability to dissect the genetics of quantitative traits related to important agronomic and physiological traits. Rapid and precise estimation of traits is essential for large-scale soybean breeding programs for genetic screening, genomic selection, and gene discovery, because selections need to be accomplished for a large number of plant populations in a short period of time (Chaudhary et al. 2015; Hou et al. 2008). In soybean, total protein and oil content can be reliably measured using near infrared (NIR) methods (Baianu et al. 2012). However, measurement of specific amino acids, carbohydrate content, and fatty acids requires, high-resolution nuclear magnetic resonance (HR-NMR), high performance liquid chromatography (HPLC) or gas chromatography (GC) platforms and wet chemistry methods (Ravindran et al. 2009). These platforms/methods are expensive and low throughput. Kovalenko et al. (2006) used NIR to estimate amino acids in soybean seeds, however, several amino acids were under predicted. Nevertheless, Panthee and Pantalone (2006) identified soybean line TN04-5321 with elevated cysteine concentration, among a population of 101 RIL, using NIR and confirming the significant increase in cysteine with wet chemistry samples from six environments. Baianu et al. (2012) reported the HR-NMR determinations, calibrations, and methodologies of amino acid profiles of proteins from whole soybean seeds, without protein extraction from the seed. Additionally, they reported a high (99%) linear correlation between NMR and NIR and suggested that both techniques are suitable for the non-destructive, practical determination of both oil and protein content of soybean seed. Recent developments in high-resolution Fourier transform (FT-NIR) extend the NIR sensitivity range to the picogram level and such developments are potentially important for breeding and biotechnology applications that require rapid and precise analyses, such as those concerned with high-content microarrays in genomic and proteomic research. For soybean seed composition, various manufacturers such as Perten and Foss make NIR instruments used by soybean research community in the US. It has been reported that different platforms give varying estimates but that can be improved using common seed source, sample calibration, multivariate analysis, and equation correction using different statistical models. Moreover, the refinement of non-destructive and single seed calibration, and predictions are desirable for the mapping applications where seed cannot be sacrificed.

#### Induced mutation and transgenic approaches

Improvement of protein and amino acid profiles have been a major, long-term goal of soybean geneticists/breeders, however, the narrow genetic base and genome complexity of soybean limit the efforts for genetic mapping and genomic improvement. Mutagenized populations (physical, chemical, transposon tagging or transformation-induced mutagens) have been useful in crop improvement (Bolon et al. 2014; Hancock et al. 2011; Shi et al. 2015). These mutagens are important for introducing genetic variation to be used in trait discovery, functional validation, and breeding. Fortunately, larger scale mutant screening is now very efficient due to NGS technologies coupled with comparative genomic hybridization (CGH) to detect copy number variations, polymorphism, and structural variations in the genome (Bolon et al. 2014). In soybean, mutations for several agronomic traits, including cyst nematode resistance (Shi et al. 2015), oleic acid (Sandhu et al. 2007), chlorophyll deficiency (Campbell et al. 2015), oil (Bolon et al. 2014; Schmidt and Herman 2008), stearic acid (Gillman et al. 2014), and nodulation (Men et al. 2002) were discovered using induced mutation. The mutant resources from the large-scale soybean fast neutron mutation project, supported by the National Science Foundation, are available for the soybean community and can be utilized for soybean meal improvement and other traits (Bolon et al. 2011, 2014) (http://www.soybase.org/mutants/about.php). Nevertheless, it is important to take into account that induced mutations can also make undesirable changes in the genome leading to yield drag and other detrimental effects (Gillman et al. 2014; Sandhu et al. 2007).

Similarly, genetic engineering approaches (e.g., overexpression, gene knockdown using RNAi, CRISPR-Cas9) have greater applications in trait discovery, functional characterization, and crop improvement. These approaches have been utilized in the past to improve seed composition traits in soybean with varying success rates (Chaudhary et al. 2015; Li et al. 2015; Tabe and Higgins 1998). The success of transgenic approaches depends upon transgene expression, effects on phenotype, genetic backgrounds as well as phenotypic transferability from in vitro condition to the field. These transgenic approaches offer new possibilities for engineering soybean seed protein and amino acids for feed and food.

## Strategic outlook and future prospects

Seed protein is considered as the most valuable trait of soybean; however, limited success has been achieved in terms of scientific understanding and improvement of this trait. Additionally, only few of the proteins and oil QTLs reported to date have been functionally characterized. Breeding for soybean seed composition traits is a complicated process; fortunately, ample genomic resources and tools are now available to soybean researchers for dissection of seed composition traits (Bandillo et al. 2015; Phansak et al. 2016). The combination of conventional breeding strategy and genomic approaches will help to identify genomic loci, haplotypes, and genetic markers aiding in breeding for improvement of seed composition traits. Once genome-wide allelic and haplotype data are available on important breeding lines and haplotype-trait associations are established for protein, oil, and amino acid traits, it may be possible for soybean breeders to undertake breeding-by-design approaches. As discussed, the major protein QTL on Chr. 20 shows distinct haplotypes among different populations and these haplotypes/alleles may facilitate breeders to select parental lines and consider them for crossing schemes or introgression into locally adapted superior yielding cultivars (Phansak et al. 2016).

While generating genomic resources becomes routine and relatively easy to handle, soybean researchers also need to develop precise and cost-effective phenotyping for several seed composition traits, especially for amino acids, and to develop breeder friendly databases and decision making tools to ensure undertaking genomics-assisted breeding approaches. An understanding of the networks of interactions between different seed traits during seed development is also crucial. In addition to these analytical needs, we recognize several issues, including protein stability under cooler temperatures, protein increase without yield drag, pleiotropic effects, and background/allelic effects. These issues could be addressed via screening diverse germplasm and selecting for environmental stability, considering wild soybean alleles for introgression, undertaking GAB (e.g., GWAS, haplotype blocks, synteny analysis, NIL etc.), precise and high-throughput phenotyping, and considering mutation breeding and genome engineering/editing. Integrating these aspects and comprehensive learning will extend our current genetic and genomic portfolio far beyond that of traditional breeding. Soybean meal will, however, only remain a leading source of protein if its quality (essential amino acids) can exceed that of other competing meal sources. To accelerate this demand on a larger scale, an integration of breeding and genomics-assisted approaches is required at a greater extent than currently being implemented.

### **Author contribution statement**

GP, TV, VP, QS, PC, GS, and TC have performed the ground work and inquest for all the relevant literature and written the review. GP performed haplotype and synteny analysis. RM and HTN conceptualized and edited the review.

## Electronic supplementary material

Below is the link to the electronic supplementary material.

Supplementary material 1 (DOCX 14 kb)

Supplementary material 2 (DOCX 35 kb)

Supplementary material 3 (DOCX 26 kb)

Supplementary material 4 (DOCX 20 kb)

**Supplementary Figure 1:** Syntenic analysis of major protein QTL on Chr. 20 (Gm20: 29803077–31462800) (JPEG 1461 kb)
