# Supplementary material for: Molecular mapping and genomics of soybean seed protein: a review and perspective for the future
Source: Theor Appl Genet. 2017 Aug 11;130(10):1975–91. doi: 10.1007/s00122-017-2955-8 (PMC5606949; doi:10.1007/s00122-017-2955-8)
Supplement: Supplementary file 1 — Supplementary material 1 (DOCX 14 kb) [file 122_2017_2955_MOESM1_ESM.docx]

**Supplementary Table 1:** Comparative value of seed protein content in various leguminous crops.

| ***Species*** | ***Protein*** | ***Reference*** |
| --- | --- | --- |
| *Arachis hypogaea* | 26.5 | Grosso and Guzman (1995) |
| *Cicer arietinum* | 22.6 | Upadhyaya et. al. (2016) |
| *Glycine max* | 40.5 | Schmutz et. al. (2010) |
| *Glycine soja* | 45.8 | Schmutz et. al. (2010) |
| *Lens esculenta* | 29.3 | Monti and Grillo (1983) |
| *Lupinus albus* | 38.7 | Monti and Grillo (1983) |
| *Phaseolus vulgaris* | 39.4 | Monti and Grillo (1983) |
| *Pisum sativum* | 39.7 | Monti and Grillo (1983) |
| *Vicia faba* | 37 | Monti and Grillo (1983) |

**References:**

Grosso NR, Guzman CA (1995) Chemical composition of aboriginal peanut (*Arachis hypogaea* L.) seeds from Peru. J Agri Food Chem 43(1):102-5.

Monti LM, Grillo S (1983). Legume seed improvement for protein content and quality. Plant Foods for Human Nutrition. 1 ;32(3):253-66.

Upadhyaya HD, Bajaj D, Narnoliya L, Das S, Kumar V, Gowda CL, Sharma S, Tyagi AK, Parida SK (2016). Genome-wide scans for delineation of candidate genes regulating seed-protein content in chickpea. Front Plant Sci. 7. <https://doi.org/10.3389/fpls.2016.00302>

Schmutz J, Cannon SB, Schlueter J, Ma J, Mitros T, Nelson W, Hyten DL, Song Q, Thelen JJ, Cheng J (2010) Genome sequence of the palaeopolyploid soybean. Nature 463:178-183
