# Supplementary material for: Molecular mapping and genomics of soybean seed protein: a review and perspective for the future
Source: Theor Appl Genet. 2017 Aug 11;130(10):1975–91. doi: 10.1007/s00122-017-2955-8 (PMC5606949; doi:10.1007/s00122-017-2955-8)
Supplement: Supplementary file 2 — Supplementary material 2 (DOCX 35 kb) [file 122_2017_2955_MOESM2_ESM.docx]

**Supplementary Table 2:** Details of QTL mapping studies performed for seed protein in soybean. Data collected from http://soybase.org/ on Feb. 01-2017.

| **QTL Name** | **LG** | **Start** | **End** | **Population types** | **Loci associated with the QTL** | **Parent 1** | **Parent 2** | **Pop. size:** | **References** |
| --- | --- | --- | --- | --- | --- | --- | --- | --- | --- |
| Seed protein 3-2 | B1 | 28.17 | 30.17 | F2:4, F2:5, F2:6 | A109_1 | M82806 | HHP | 677 | Brummer et al. 1997 Mapping QTL for seed protein and oil content in eight soybean populations  Crop Sci. 1997, 37(2):370-378 |
| Seed protein 3-3 | C1 | 89.7 | 91.7 | F2:4, F2:5, F2:6 | A063_1 | M82806 | HHP | 677 |  |
| Seed protein 3-4 | D1a | 5.43 | 7.43 | F2:4, F2:5, F2:6 | A398_1 | M82806 | HHP | 677 |  |
| Seed protein 3-5 | D1a | 39.8 | 41.8 | F2:4, F2:5, F2:6 | A691_1 | M82806 | HHP | 677 |  |
| Seed protein 3-6 | E | 29.88 | 31.88 | F2:4, F2:5, F2:6 | B174_1 | M82806 | HHP | 677 |  |
| Seed protein 3-7 | F | 46.63 | 48.63 | F2:4, F2:5, F2:6 | K002_1 | M82806 | HHP | 677 |  |
| Seed protein 3-10 | G | 96.24 | 98.24 | F2:4, F2:5, F2:6 | A235_1 | M82806 | HHP | 677 |  |
| Seed protein 3-8 | G | 66.53 | 68.53 | F2:4, F2:5, F2:6 | A816_1 | M82806 | HHP | 677 |  |
| Seed protein 3-9 | G | 66.7 | 68.7 | F2:4, F2:5, F2:6 | A890_1 | M82806 | HHP | 677 |  |
| Seed protein 3-11 | H | 32.17 | 34.17 | F2:4, F2:5, F2:6 | A069_1 | M82806 | HHP | 677 |  |
| Seed protein 3-12 | I | 31.42 | 33.42 | F2:4, F2:5, F2:6 | A144_1 | M82806 | HHP | 677 |  |
| Seed protein 3-1 | A2 | 131.3 | 133.31 | F2:4, F2:5, F2:6 | A505_1 | M82806 | HHP | 677 |  |
| Seed protein 16-1 | B1 | 35.48 | 37.48 | F4:6 | Satt251 | Essex | Essex |  | Chapman et al. 2003 Quantitative trait loci for agronomic and seed quality traits in an F-2 and F-4:6 soybean population  Euphytica 2003, 129(3):387-393 |
| Seed protein 16-2 | L | 106.2 | 108.24 | F4:6 | Satt373 | Essex | Essex |  |  |
| Seed protein 27-1 | D1b | 70.65 | 75.66 | F2:10 RIL | Sat_135 Satt537 | Charleston | Dongnong 594 | 154 | Chen et al. 2007 QTL Analysis of Major Agronomic Traits in Soybean  Ag. Sci. in China 2007, 6(4):399-405 |
| Seed protein 27-2 | E | 44.93 | 45.4 | F2:10 RIL | Satt151 Satt263 | Charleston | Dongnong 594 | 154 |  |
| Seed protein 27-3 | E | 44.93 | 45.77 | F2:10 RIL | Satt117 Satt151 | Charleston | Dongnong 594 | 154 |  |
| Seed protein 27-4 | N | 91.55 | 114.55 | F2:10 RIL | Satt257 | Charleston | Dongnong 594 | 154 |  |
| Seed protein 27-5 | O | 58.4 | 106.02 | F2:10 RIL | Satt173 Satt581 | Charleston | Dongnong 594 | 154 |  |
| Seed protein 15-1 | I | 36.4 | 36.94 | F5-derived RILs | Satt239 | A3733 | PI437088A |  | Chung et al. 2003 The seed protein, oil, and yield QTL on soybean linkage group I  Crop Sci. 2003, 43(3):1053-1067 |
| Seed protein 13-2 | C2 | 121 | 123.02 | F2 | Sct_028 | Ma.Belle | Proto |  | Csanadi et al. 2001 Seed quality QTLs identified in a molecular map of early maturing soybean  Theor. Appl. Genet. 2001, 103(6-7):912-919 |
| Seed protein 13-1 | D1a | 76.5 | 78.5 | F2 | Satt077 | Ma.Belle | Proto |  |  |
| Seed protein 13-4 | K | 103.8 | 105.79 | F2 | Satt196 | Ma.Belle | Proto |  |  |
| Seed protein 13-3 | M | 32.5 | 34.5 | F2 | Satt567 | Ma.Belle | Proto |  |  |
| Seed protein 1-6 | B2 | 32.13 | 34.13 | F2:3 | A242_1 | A81356022 | PI468916 | 60 | Diers et al. 1992C RFLP analysis of soybean seed protein and oil content  Theor. Appl. Genet. 1992, 83(5):608-612 |
| Seed protein 1-5 | E | 5.3 | 7.3 | F2:3 | SAC7_1 | A81356022 | PI468916 | 60 |  |
| Seed protein 1-8 | G | 88.97 | 90.97 | F2:3 | A245_2 | A81356022 | PI468916 | 60 |  |
| Seed protein 1-1 | I | 37.06 | 39.06 | F2:3 | K011_1 | A81356022 | PI468916 | 60 |  |
| Seed protein 1-2 | I | 38.4 | 40.4 | F2:3 | A407_1 | A81356022 | PI468916 | 60 |  |
| Seed protein 1-3 | I | 31.42 | 33.42 | F2:3 | A144_1 | A81356022 | PI468916 | 60 |  |
| Seed protein 1-4 | I | 31.4 | 33.4 | F2:3 | A688_1 | A81356022 | PI468916 | 60 |  |
| Seed protein 1-7 | L | 35.7 | 37.7 | F2:3 | A023_1 | A81356022 | PI468916 | 60 |  |
| Seed protein 33-2 | F | 71.41 | 77.69 | F4:6 RIL | Satt510 - Satt335 | OAC Wallace | OAC Glencoe | 203 | Eskandari et al. 2013b Genetic control of soybean seed oil: II. QTL and genes that increase oil concentration without decreasing protein or with increased seed yield  Theor. Appl. Genet. 2013 126(6):1677-1687 |
| Seed protein 33-3 | K | 71.41 | 77.69 | F4:6 RIL | Satt001 - Satt273 | OAC Wallace | OAC Glencoe | 203 |  |
| Seed protein 33-4 | L | 14.03 | 27.92 | F4:6 RIL | Satt182 - Satt523 | OAC Wallace | OAC Glencoe | 203 |  |
| Seed protein 33-5 | M | 50.09 | 60.07 | F4:6 RIL | Satt323 - Satt463 | OAC Wallace | OAC Glencoe | 203 |  |
| Seed protein 33-1 | H | 81.04 | 89.51 | F4:6 RIL | Satt317- Satt302 | OAC Wallace | OAC Glencoe | 203 |  |
| cqSeed protein-001 | E | 29.89 | 31.89 | F2 derived | A454_1 | PI 97100 | Coker 237 | 176 | Fasoula et al. 2004 Validation and Designation of Quantitative Trait Loci for Seed Protein, Seed Oil, and Seed Weight from Two Soybean Populations  Crop Sci. 2004, 44(4):1218-1225 |
| cqSeed protein-003 | I | 35.4 | 37.4 | F2 derived | Satt496 | PI 97100 | Coker 237 | 176 |  |
| cqSeed protein-002 | N/A | N/A | N/A | F2 derived | A132_4 | PI 97100 | Coker 237 | 176 |  |
| Seed protein 25-1 | B1 | 46.38 | 58.91 | F2:7:10 RIL | A118_1 Satt197 Satt560 | Kefeng No. 1 | 1138-2 | 201 | Gai et al. 2007 A comparative study on segregation analysis and QTL mapping of quantitative traits in plants-with a case in soybean  Front. of Ag. in China. 2007, 1(1):1-7 |
| Seed protein 25-2 | B1 | 58.91 | 59.1 | F2:7:10 RIL | A118_1 A520_1 | Kefeng No. 1 | 1138-2 | 201 |  |
| Seed protein 25-3 | D1b | 7.63 | 9.63 | F2:7:10 RIL | A725_1 | Kefeng No. 1 | 1138-2 | 201 |  |
| Seed protein 24-1 | C2 | 117.8 | 121.3 | F6,8RIL | Satt202 Satt277 | Essex | Williams | 131 | Hyten et al. 2004A Seed quality QTL in a prominent soybean population  Theor. Appl. Genet. 2004, 109(3):552-561 |
| Seed protein 24-2 | F | 79.7 | 99.7 | F6,8RIL | Satt144 Satt335 | Essex | Williams | 131 |  |
| Seed protein 24-3 | K | 5.8 | 25.8 | F6,8RIL | Satt102 Satt539 | Essex | Williams | 131 |  |
| Seed protein 24-4 | M | 35.85 | 50.1 | F6,8RIL | Satt463 Satt540 | Essex | Williams | 131 |  |
| Seed protein 21-1 | A2 | 144.6 | 146.57 | F5 | Satt409 | BSR 101 | LG82-8379 | 167 | Kabelka et al. 2004 Putative alleles for increased yield from soybean plant introductions  Crop Sci. 2004, 44(3):784-791 |
| Seed protein 21-8 | B2 | 54.2 | 56.2 | F5 | Satt168 | BSR 101 | LG82-8379 | 167 |  |
| Seed protein 21-2 | C1 | 122.8 | 124.79 | F5 | Satt338 | BSR 101 | LG82-8379 | 167 |  |
| Seed protein 21-3 | C2 | 97.07 | 99.07 | F5 | Satt363 | BSR 101 | LG82-8379 | 167 |  |
| Seed protein 21-4 | D1b | 36.07 | 38.07 | F5 | Satt157 | BSR 101 | LG82-8379 | 167 |  |
| Seed protein 21-6 | F | 70.41 | 72.41 | F5 | Satt510 | BSR 101 | LG82-8379 | 167 |  |
| Seed protein 21-10 | H | 85.49 | 86.49 | F5 | Satt142 | BSR 101 | LG82-8379 | 167 |  |
| Seed protein 21-11 | M | 129.8 | 131.76 | F5 | Satt308 | BSR 101 | LG82-8379 | 167 |  |
| Seed protein 21-9 | N | 74.91 | 76.91 | F5 | Satt339 | BSR 101 | LG82-8379 | 167 |  |
| Seed protein 21-5 | O | 4.44 | 6.44 | F5 | Satt358 | BSR 101 | LG82-8379 | 167 |  |
| Seed protein 4-1 | C1 | 120 | 122 | F4-derived | gc197_1 | Young | PI416937 | 120 | Lee et al. 1996C RFLP loci associated with soybean seed protein and oil content across populations and locations  Theor. Appl. Genet. 1996, 93(5-6):649-657 |
| Seed protein 5-1 | E | 29.9 | 31.9 | F2-derived | A454_1 | PI97100 | Coker237 | 111 |  |
| Seed protein 5-2 | H | 85.8 | 87.8 | F2-derived | A566_2 | PI97100 | Coker237 | 111 |  |
| Seed protein 5-3 | K | 52.9 | 54.9 | F2-derived | A065_3 | PI97100 | Coker237 | 111 |  |
| Seed protein 5-4 | K | 30.75 | 32.75 | F2-derived | R051_2 | PI97100 | Coker237 | 111 |  |
| Seed protein 5-5 | N/A | N/A | N/A | F2-derived | Q043_1 | PI97100 | Coker237 | 111 |  |
| Seed protein 5-6 | N/A | N/A | N/A | F2-derived | A132_4 | PI97100 | Coker237 | 111 |  |
| Seed protein 4-10 | B2 | 42.6 | 45.6 | F4-derived | B142_1 | Young | PI416937 | 120 |  |
| Seed protein 4-11 | B2 | 28.19 | 30.19 | F4-derived | A352_1 | Young | PI416937 | 120 |  |
| Seed protein 4-2 | C1 | 125.5 | 127.5 | F4-derived | EV3_1 | Young | PI416937 | 120 |  |
| Seed protein 4-3 | C1 | 96 | 98 | F4-derived | A338_2 | Young | PI416937 | 120 |  |
| Seed protein 4-4 | C1 | 20.04 | 22.04 | F4-derived | A463_1 | Young | PI416937 | 120 |  |
| Seed protein 4-13 | E | 44 | 46 | F4-derived | cr274_1 | Young | PI416937 | 120 |  |
| Seed protein 4-5 | E | 25.02 | 27.02 | F4-derived | A517_1 | Young | PI416937 | 120 |  |
| Seed protein 4-6 | E | 26.3 | 28.3 | F4-derived | cr167_1 | Young | PI416937 | 120 |  |
| Seed protein 4-7 | J | 26.63 | 28.63 | F4-derived | B166_1 | Young | PI416937 | 120 |  |
| Seed protein 4-8 | N | 29.35 | 31.35 | F4-derived | A071_2 | Young | PI416937 | 120 |  |
| Seed protein 4-9 | N | 33.4 | 35.4 | F4-derived | gc34_2 | Young | PI416937 | 120 |  |
| Seed protein 4-12 | N/A | N/A | N/A | F4-derived | A199_3 | Young | PI416937 | 120 |  |
| Seed protein 28-4 | B2 | 77.38 | 94.48 | F13 | Satt063 Satt070 | Jindou 23 | Huibuzhi | 474 | Liang et al. 2010 QTL Mapping of Isoflavone, Oil and Protein Contents in Soybean (Glycine max L. Merr.)  Ag. Sci. China 2010, 9(8):1108-1116 |
| Seed protein 28-1 | C2 | 112.9 | 114.9 | F13 | Satt557 Satt100 | Jindou 23 | Huibuzhi | 474 |  |
| Seed protein 28-2 | G | 62.16 | 67.16 | F13 | Satt199 Satt012 | Jindou 23 | Huibuzhi | 474 |  |
| Seed protein 28-5 | G | 1.84 | 17.64 | F13 | Satt275 Satt038 | Jindou 23 | Huibuzhi | 474 |  |
| Seed protein 28-3 | H | 30.95 | 46.95 | F13 | Satt568 Satt442 | Jindou 23 | Huibuzhi | 474 |  |
| Seed protein 34-1 | A1 | 92.45 | 95.95 | F2:9 | Satt211 | ZDD09454 | Yudou12 | 212 | Lu et al. 2012 Identification of the quantitative trait loci (QTL) underlying water soluble protein content in soybean  Theor. Appl. Gen. 2012, Online First |
| Seed protein 34-4 | A2 | 52.57 | 81.01 | F2:9 | Sat_115 - GMENOD2B | ZDD09454 | Yudou12 | 212 |  |
| Seed protein 34-5 | A2 | 52.57 | 54.57 | F2:9 | Sat_215 - GMENOD2B | ZDD09454 | Yudou12 | 212 |  |
| Seed protein 34-7 | B1 | 24.66 | 35 | F2:9 | Sat_156 | ZDD09454 | Yudou12 | 212 |  |
| Seed protein 34-2 | C2 | 69.69 | 90.93 | F2:9 | Sat_213 - GMAC7L | ZDD09454 | Yudou12 | 212 |  |
| Seed protein 34-9 | G | 56.18 | 61.41 | F2:9 | Sat_094, Satt564, Sat_260 | ZDD09454 | Yudou12 | 212 |  |
| Seed protein 34-8 | H | 53.34 | 58.91 | F2:9 | Satt541, Satt469 | ZDD09454 | Yudou12 | 212 |  |
| Seed protein 34-11 | I | 35.16 | 50.11 | F2:9 | AB002807, Satt270 | ZDD09454 | Yudou12 | 212 |  |
| Seed protein 34-6 | K | 67.9 | 80.68 | F2:9 | Satt475 | ZDD09454 | Yudou12 | 212 |  |
| Seed protein 34-10 | L | 29.19 | 33.19 | F2:9 | Satt143, Sat_195 | ZDD09454 | Yudou12 | 212 |  |
| Seed protein 34-3 | M | 130.8 | 136.75 | F2:9 | Satt336, Satt308 | ZDD09454 | Yudou12 | 212 |  |
| Seed protein 2-1 | A1 | 92.59 | 94.59 | F7-derived RIL | T155_1 | Noir 1 | Minsoy | 284 | Mansur et al. 1996 Genetic mapping of agronomic traits using recombinant inbred lines of soybean.  Crop Sci. 1996, 36(5):1327-1336 |
| Seed protein 2-3 | A1 | 29.28 | 31.28 | F7-derived RIL | A329_2 | Noir 1 | Minsoy | 284 |  |
| Seed protein 2-2 | L | 91 | 93 | F7-derived RIL | Satt006 | Noir 1 | Minsoy | 284 |  |
| Seed protein 9-1 | A1 | 92.59 | 94.59 |  | T155_1 | Minsoy | Noir 1 |  | Orf et al. 1999A Genetics of soybean agronomic traits: I. Comparison of three related recombinant inbred populations  Crop Sci. 1999, 39(6):1642-1651 |
| Seed protein 7-2 | C1 | 64.08 | 66.08 |  | Satt578 | Minsoy | Archer |  |  |
| Seed protein 9-2 | C1 | 9.34 | 11.34 |  | SOYGPATR | Minsoy | Noir 1 |  |  |
| Seed protein 8-1 | L | 65.5 | 67.5 |  | Satt166 | Noir 1 | Archer |  |  |
| Seed protein 7-1 | M | 37.98 | 39.98 |  | R079_1 | Minsoy | Archer |  |  |
| Seed protein 29-1 | C2 | 113 | 114.95 | F4:7 RIL | Satt100 | OAC Millennium | Heinong 38 | 98 | Palomeque et al. 2009b QTL in mega-environments: II. Agronomic trait QTL co-localized with seed yield QTL detected in a population derived from a cross of high-yielding adapted x high-yielding exotic soybean lines  Theor. Appl. Genet. 2009, 119(3):429-436 |
| Seed protein 31-4 | C2 | 41.36 | 43.36 | F4:5 RIL | Satt520 | X3145-B-B-3-15 | AC Brant | 201 | Pandurangan et al. 2012 Relationship between asparagine metabolism and protein concentration in soybean seed  J. Exp. Bot. 2012 |
| Seed protein 31-3 | D1a | 76.48 | 78.48 | F4:5 RIL | Satt077 | X3145-B-B-3-15 | AC Brant | 201 |  |
| Seed protein 31-2 | E | 2.3 | 4.3 | F4:5 RIL | Satt575 | X3145-B-B-3-15 | AC Brant | 201 |  |
| Seed protein 31-1 | I | 35.4 | 37.4 | F4:5 RIL | Satt496 | X3145-B-B-3-15 | AC Brant | 201 |  |
| Seed protein 20-1 | G | 11.74 | 13.74 | F6 | Satt570 | N87-984-16 | TN93-99 | 101 | Panthee et al. 2005 Quantitative Trait Loci for Seed Protein and Oil Concentration, and Seed Size in Soybean  Crop Sci. 2005, 45(5):2015-2022 |
| Seed protein 6-2 | F | 104.8 | 106.84 | F2:3 | B148_1 | Peking | Essex | 200 | Qiu et al. 1999 RFLP markers associated with soybean cyst nematode resistance and seed composition in a 'Peking' x 'Essex' population.  Theor. Appl. Genet. 1999, 98(3-4):356-364 |
| Seed protein 6-1 | H | 123.1 | 125.05 | F2:3 | B072_1 | Peking | Essex | 200 |  |
| Seed protein 26-1 | A2 | 53.91 | 55.91 | F6 and F7 RIL | Satt187 | RG10 | OX948 | 169 | Reinprecht et al. 2006 Seed and agronomic QTL in low linolenic acid, lipoxygenase-free soybean (Glycine max (L.) Merrill) germplasm.  Genome 2006, 49(12):1510-1527 |
| Seed protein 26-6 | B1 | 99.87 | 101.87 | F6 and F7 RIL | Sat_123 | RG10 | OX948 | 169 |  |
| Seed protein 26-10 | B2 | 92.48 | 94.48 | F6 and F7 RIL | Satt063 | RG10 | OX948 | 169 |  |
| Seed protein 26-7 | C2 | 106.6 | 108.58 | F6 and F7 RIL | Satt277 | RG10 | OX948 | 169 |  |
| Seed protein 26-2 | D2 | 78.23 | 80.23 | F6 and F7 RIL | Satt389 | RG10 | OX948 | 169 |  |
| Seed protein 26-11 | F | 129.6 | 131.63 | F6 and F7 RIL | Sat_090 | RG10 | OX948 | 169 |  |
| Seed protein 26-13 | F | 2.35 | 4.35 | F6 and F7 RIL | Satt569 | RG10 | OX948 | 169 |  |
| Seed protein 26-12 | G | 3.53 | 5.53 | F6 and F7 RIL | Satt309 | RG10 | OX948 | 169 |  |
| Seed protein 26-14 | G | 42.38 | 44.38 | F6 and F7 RIL | Satt394 | RG10 | OX948 | 169 |  |
| Seed protein 26-8 | G | 42.38 | 44.38 | F6 and F7 RIL | Satt394 | RG10 | OX948 | 169 |  |
| Seed protein 26-3 | I | 17.5 | 19.5 | F6 and F7 RIL | Satt571 | RG10 | OX948 | 169 |  |
| Seed protein 26-4 | I | 20.9 | 22.9 | F6 and F7 RIL | Satt419 | RG10 | OX948 | 169 |  |
| Seed protein 26-5 | I | 49.11 | 51.11 | F6 and F7 RIL | Satt270 | RG10 | OX948 | 169 |  |
| Seed protein 26-9 | I | 85.73 | 87.73 | F6 and F7 RIL | Satt162 | RG10 | OX948 | 169 |  |
| Seed protein 35-1 | C2 | 106.6 | 108.58 | F4:7 RIL | Satt277 | OAC Millenium | Heinong 38 | 92 | Rossi et al. 2013 Genetic basis of soybean adaptation to North American vs. Asian mega-environments in two independent populations from Canadian x Chinese crosses  Theor. Appl. Genet. 2013, 126(7):1809-1823 |
| Seed protein 35-2 | C2 | 113 | 114.95 | F4:7 RIL | Satt100 | OAC Millenium | Heinong 38 | 92 |  |
| Seed protein 35-3 | I | 83.73 | 85.73 | F4:7 RIL | Satt162 | OAC Millenium | Heinong 38 | 92 |  |
| Seed protein 35-4 | K | 55.61 | 57.61 | F4:7 RIL | Satt273 | OAC Millenium | Heinong 38 | 92 |  |
| Seed protein 10-1 | I | 34.35 | 36.35 | Backcross3 | Satt127 | G.max | G.soja |  | Sebolt et al. 2000 Analysis of a quantitative trait locus allele from wild soybean that increases seed protein concentration in soybean  Crop Sci. 2000, 40(5):1438-1444 |
| Seed protein 11-1 | I | 31.42 | 33.42 | BC3F4-derived | A144_1 | Parker | G.soja |  |  |
| Seed protein 12-1 | A1 | 93.92 | 95.92 | F7:11 RIL | B170_1 | Minsoy | Noir 1 |  | Specht et al. 2001 Soybean Response to Water:A QTL Analysis of Drought Tolerance  Crop Sci. 2001, 41(2):493-509 |
| Seed protein 12-2 | C1 | 32.3 | 34.3 | F7:11 RIL | K001_1 | Minsoy | Noir 1 |  |  |
| Seed protein 12-3 | K | 39.86 | 41.86 | F7:11 RIL | Satt178 | Minsoy | Noir 1 |  |  |
| Seed protein 12-4 | M | 32.5 | 34.5 | F7:11 RIL | Satt567 | Minsoy | Noir 1 |  |  |
| Seed protein 12-5 | O | 70.1 | 72.1 | F7:11 RIL | Satt478 | Minsoy | Noir 1 |  |  |
| Seed protein 19-1 | C1 | 47.08 | 75.08 | RI | L92_1 - Satt578 | Minsoy | Archer | 108 | Stombaugh et al. 2004 Quantitative Trait Loci Associated with Cell Wall Polysaccharides in Soybean Seed  Crop Sci. 2004, 44:2101-2106 |
| Seed protein 17-5 | A1 | 90.3 | 94.3 | F7:8 RIL | A104_1 | Misuzudaizu | Moshidou Gong 503 |  | Tajuddin et al. 2003 Analysis of quantitative trait loci for protein and lipid contents in soybean seeds using recombinant inbred lines  Breed. Sci. 2003, 53(2):133-140 |
| Seed protein 17-4 | A2 | 48.5 | 49.5 | F7:8 RIL | I | Misuzudaizu | Moshidou Gong 503 |  |  |
| Seed protein 30-4 | A2 | 42.98 | 54.71 | F8 RIL | I | Misuzudaizu | Moshidou Gong 503 | 156 |  |
| Seed protein 30-5 | C2 | 29.92 | 50.65 | F8 RIL | Satt281- Satt520 | Misuzudaizu | Moshidou Gong 503 | 156 |  |
| Seed protein 17-2 | D2 | 105.5 | 109.49 | F8 RIL | Satt310 | Misuzudaizu | Moshidou Gong 503 |  |  |
| Seed protein 30-2 | D2 | 99.02 | 115.64 | F8 RIL | Satt310 | Misuzudaizu | Moshidou Gong 503 | 156 |  |
| Seed protein 17-3 | E | 18.8 | 20 | F7:8 RIL | Satt384 | Misuzudaizu | Moshidou Gong 503 |  |  |
| Seed protein 30-3 | E | 13.73 | 24.85 | F8 RIL | Satt384 | Misuzudaizu | Moshidou Gong 503 | 156 |  |
| Seed protein 30-10 | G | 106.6 | 112.34 | F8 RIL | A378_1 | Misuzudaizu | Moshidou Gong 503 | 156 |  |
| Seed protein 17-1 | I | 35.94 | 37.94 | F7:8 RIL | Satt239 - | Misuzudaizu | Moshidou Gong 503 |  |  |
| Seed protein 30-1 | I | 25.68 | 48.18 | F8 RIL | Satt367, Satt127, Satt239 | Misuzudaizu | Moshidou Gong 503 | 156 |  |
| Seed protein 17-6 | L | 52.14 | 56.14 | F7:8 RIL | Satt156 | Misuzudaizu | Moshidou Gong 503 |  |  |
| Seed protein 30-7 | L | 51.02 | 56.13 | F8 RIL | Satt156 | Misuzudaizu | Moshidou Gong 503 | 156 |  |
| Seed protein 30-6 | N/A | N/A | N/A | F8 RIL | 9999 | Misuzudaizu | Moshidou Gong 503 | 156 |  |
| Seed protein 30-8 | N/A | N/A | N/A | F8 RIL | 9999 | Misuzudaizu | Moshidou Gong 503 | 156 |  |
| Seed protein 30-9 | N/A | N/A | N/A | F8 RIL | 9999 | Misuzudaizu | Moshidou Gong 503 | 156 |  |
| Seed protein 14-1 | A2 | 149 | 151 | F2:3, F2:4 | Ti | M91-212006 | SZG9652 | 128 | Vollmann et al. 2002 The presence or absence of the soybean Kunitz trypsin inhibitor as a quantitative trait locus for seed protein content  Plant Breed. 2002, 121(3):272-274 |
| qProt_Gm14 | B2 | 0.6 | 7.9 | F5 | BARC-018353–BARC-056587 | Benning | Danbaekkong | 140 | Warrington, C.*, et al.* (2015) QTL for seed protein and amino acids in the Benning× Danbaekkong soybean population. *Theor. Appl. Genet.* 128, 839-850 |
| qProt_Gm15 | E | 29.2 | 38.7 | F5 | Sat_273–BARC-027786 | Benning | Danbaekkong | 140 |  |
| qProt_Gm17 | D2 | 52.6 | 60.4 | F5 | BARC-025927–Satt256 | Benning | Danbaekkong | 140 |  |
| qProt_Gm20 | I | 14.7 | 20.7 | F5 | GSM0012–Satt354 | Benning | Danbaekkong | 140 |  |
| Seed protein 32-1 | B2 | 96.91 | 98.91 | F2:7:10 RIL | Satt560 | Kefeng No. 1 | Nannong 1138-2 | 206 | Zhang et al, 2004 QTL mapping of ten agronomic traits on the soybean (Glycine max L. Merr) genetic map and their association with EST markers  Theor. Appl. Genet. 2004, 108:1131Ü1139 |
