# Supplementary material for: Molecular mapping and genomics of soybean seed protein: a review and perspective for the future
Source: Theor Appl Genet. 2017 Aug 11;130(10):1975–91. doi: 10.1007/s00122-017-2955-8 (PMC5606949; doi:10.1007/s00122-017-2955-8)
Supplement: Supplementary file 3 — Supplementary material 3 (DOCX 26 kb) [file 122_2017_2955_MOESM3_ESM.docx]

**Supplementary Table 3:** Details of QTL mapping studies performed for seed amino acids in soybean. Data collected from http://soybase.org/ on Feb. 01-2016.

| **QTL Name** | **LG** | **Start** | **End** | **Pop. Type** | **Loci associated with the QTL** | **Parent 1** | **Parent 2** | **Pop. Size** | **References** |
| --- | --- | --- | --- | --- | --- | --- | --- | --- | --- |
| [Seed Ala 1-1](http://www.soybase.org/sbt/search/search_results.php?category=QTLName&search_term=Seed+Ala+1-1) | [A1](http://www.soybase.org/cmap/cgi-bin/cmap/viewer?data_source=sbt_cmap;ref_map_set_aid=GmComposite2003&ref_map_aids=GmComposite2003_A1&comparative_map_left=GmConsensus40_A1;highlight=%22Seed+Ala+1-1%22GmComposite2003_A1) | 24.55 | 26.55 | F6 RIL | [Satt593](http://www.soybase.org/sbt/search/search_results.php?category=LocusName&search_term=Satt593) | N87-984-16 | TN93-99 | 101 | Panthee et al. 2006B Genomic regions associated with amino acid composition in soybean  Mol. Breed. 2006, 17(1):79-89 |
| [Seed Ala 1-2](http://www.soybase.org/sbt/search/search_results.php?category=QTLName&search_term=Seed+Ala+1-2) | [A2](http://www.soybase.org/cmap/cgi-bin/cmap/viewer?data_source=sbt_cmap;ref_map_set_aid=GmComposite2003&ref_map_aids=GmComposite2003_A2&comparative_map_left=GmConsensus40_A2;highlight=%22Seed+Ala+1-2%22GmComposite2003_A2) | 106.05 | 108.05 | F6 RIL | [Satt437](http://www.soybase.org/sbt/search/search_results.php?category=LocusName&search_term=Satt437) | N87-984-16 | TN93-99 | 101 |  |
| [Seed Ala 1-3](http://www.soybase.org/sbt/search/search_results.php?category=QTLName&search_term=Seed+Ala+1-3) | [B2](http://www.soybase.org/cmap/cgi-bin/cmap/viewer?data_source=sbt_cmap;ref_map_set_aid=GmComposite2003&ref_map_aids=GmComposite2003_B2&comparative_map_left=GmConsensus40_B2;highlight=%22Seed+Ala+1-3%22GmComposite2003_B2) | 54.2 | 56.2 | F6 RIL | [Satt168](http://www.soybase.org/sbt/search/search_results.php?category=LocusName&search_term=Satt168) | N87-984-16 | TN93-99 | 101 |  |
| [Seed Ala 1-4](http://www.soybase.org/sbt/search/search_results.php?category=QTLName&search_term=Seed+Ala+1-4) | [F](http://www.soybase.org/cmap/cgi-bin/cmap/viewer?data_source=sbt_cmap;ref_map_set_aid=GmComposite2003&ref_map_aids=GmComposite2003_F&comparative_map_left=GmConsensus40_F;highlight=%22Seed+Ala+1-4%22GmComposite2003_F) | 15.08 | 17.08 | F6 RIL | [Satt252](http://www.soybase.org/sbt/search/search_results.php?category=LocusName&search_term=Satt252) | N87-984-16 | TN93-99 | 101 |  |
| [Seed Ala 1-5](http://www.soybase.org/sbt/search/search_results.php?category=QTLName&search_term=Seed+Ala+1-5) | [L](http://www.soybase.org/cmap/cgi-bin/cmap/viewer?data_source=sbt_cmap;ref_map_set_aid=GmComposite2003&ref_map_aids=GmComposite2003_L&comparative_map_left=GmConsensus40_L;highlight=%22Seed+Ala+1-5%22GmComposite2003_L) | 0 | 2 | F6 RIL | [Satt495](http://www.soybase.org/sbt/search/search_results.php?category=LocusName&search_term=Satt495) | N87-984-16 | TN93-99 | 101 |  |
| [Seed Ala 1-6](http://www.soybase.org/sbt/search/search_results.php?category=QTLName&search_term=Seed+Ala+1-6) | [L](http://www.soybase.org/cmap/cgi-bin/cmap/viewer?data_source=sbt_cmap;ref_map_set_aid=GmComposite2003&ref_map_aids=GmComposite2003_L&comparative_map_left=GmConsensus40_L;highlight=%22Seed+Ala+1-6%22GmComposite2003_L) | 29.19 | 31.19 | F6 RIL | [Satt143](http://www.soybase.org/sbt/search/search_results.php?category=LocusName&search_term=Satt143) | N87-984-16 | TN93-99 | 101 |  |
| [Seed Asp 1-1](http://www.soybase.org/sbt/search/search_results.php?category=QTLName&search_term=Seed+Asp+1-1) | [A2](http://www.soybase.org/cmap/cgi-bin/cmap/viewer?data_source=sbt_cmap;ref_map_set_aid=GmComposite2003&ref_map_aids=GmComposite2003_A2&comparative_map_left=GmConsensus40_A2;highlight=%22Seed+Asp+1-1%22GmComposite2003_A2) | 35.77 | 37.77 | F6 RIL | [Satt177](http://www.soybase.org/sbt/search/search_results.php?category=LocusName&search_term=Satt177) | N87-984-16 | TN93-99 | 101 |  |
| [Seed Asp 1-2](http://www.soybase.org/sbt/search/search_results.php?category=QTLName&search_term=Seed+Asp+1-2) | [D1a](http://www.soybase.org/cmap/cgi-bin/cmap/viewer?data_source=sbt_cmap;ref_map_set_aid=GmComposite2003&ref_map_aids=GmComposite2003_D1a&comparative_map_left=GmConsensus40_D1a;highlight=%22Seed+Asp+1-2%22GmComposite2003_D1a) | 58 | 60 | F6 RIL | [Satt203](http://www.soybase.org/sbt/search/search_results.php?category=LocusName&search_term=Satt203) | N87-984-16 | TN93-99 | 101 |  |
| [Seed Asp 1-3](http://www.soybase.org/sbt/search/search_results.php?category=QTLName&search_term=Seed+Asp+1-3) | [I](http://www.soybase.org/cmap/cgi-bin/cmap/viewer?data_source=sbt_cmap;ref_map_set_aid=GmComposite2003&ref_map_aids=GmComposite2003_I&comparative_map_left=GmConsensus40_I;highlight=%22Seed+Asp+1-3%22GmComposite2003_I) | 81.77 | 83.77 | F6 RIL | [Satt292](http://www.soybase.org/sbt/search/search_results.php?category=LocusName&search_term=Satt292) | N87-984-16 | TN93-99 | 101 |  |
| [Seed Asp 1-4](http://www.soybase.org/sbt/search/search_results.php?category=QTLName&search_term=Seed+Asp+1-4) | [K](http://www.soybase.org/cmap/cgi-bin/cmap/viewer?data_source=sbt_cmap;ref_map_set_aid=GmComposite2003&ref_map_aids=GmComposite2003_K&comparative_map_left=GmConsensus40_K;highlight=%22Seed+Asp+1-4%22GmComposite2003_K) | 103.79 | 106.79 | F6 RIL | [Satt196](http://www.soybase.org/sbt/search/search_results.php?category=LocusName&search_term=Satt196) | N87-984-16 | TN93-99 | 101 |  |
| [Seed Glu 1-1](http://www.soybase.org/sbt/search/search_results.php?category=QTLName&search_term=Seed+Glu+1-1) | [A2](http://www.soybase.org/cmap/cgi-bin/cmap/viewer?data_source=sbt_cmap;ref_map_set_aid=GmComposite2003&ref_map_aids=GmComposite2003_A2&comparative_map_left=GmConsensus40_A2;highlight=%22Seed+Glu+1-1%22GmComposite2003_A2) | 35.77 | 37.77 | F6 RIL | [Satt177](http://www.soybase.org/sbt/search/search_results.php?category=LocusName&search_term=Satt177) | N87-984-16 | TN93-99 | 101 |  |
| [Seed Glu 1-2](http://www.soybase.org/sbt/search/search_results.php?category=QTLName&search_term=Seed+Glu+1-2) | [D1a](http://www.soybase.org/cmap/cgi-bin/cmap/viewer?data_source=sbt_cmap;ref_map_set_aid=GmComposite2003&ref_map_aids=GmComposite2003_D1a&comparative_map_left=GmConsensus40_D1a;highlight=%22Seed+Glu+1-2%22GmComposite2003_D1a) | 58 | 60 | F6 RIL | [Satt203](http://www.soybase.org/sbt/search/search_results.php?category=LocusName&search_term=Satt203) | N87-984-16 | TN93-99 | 101 |  |
| [Seed Glu 1-3](http://www.soybase.org/sbt/search/search_results.php?category=QTLName&search_term=Seed+Glu+1-3) | [D1b](http://www.soybase.org/cmap/cgi-bin/cmap/viewer?data_source=sbt_cmap;ref_map_set_aid=GmComposite2003&ref_map_aids=GmComposite2003_D1b&comparative_map_left=GmConsensus40_D1b;highlight=%22Seed+Glu+1-3%22GmComposite2003_D1b) | 115.34 | 117.34 | F6 RIL | [Satt274](http://www.soybase.org/sbt/search/search_results.php?category=LocusName&search_term=Satt274) | N87-984-16 | TN93-99 | 101 |  |
| [Seed Glu 1-4](http://www.soybase.org/sbt/search/search_results.php?category=QTLName&search_term=Seed+Glu+1-4) | [J](http://www.soybase.org/cmap/cgi-bin/cmap/viewer?data_source=sbt_cmap;ref_map_set_aid=GmComposite2003&ref_map_aids=GmComposite2003_J&comparative_map_left=GmConsensus40_J;highlight=%22Seed+Glu+1-4%22GmComposite2003_J) | 10.74 | 12.74 | F6 RIL | [Satt249](http://www.soybase.org/sbt/search/search_results.php?category=LocusName&search_term=Satt249) | N87-984-16 | TN93-99 | 101 |  |
| [Seed Glu 1-5](http://www.soybase.org/sbt/search/search_results.php?category=QTLName&search_term=Seed+Glu+1-5) | [L](http://www.soybase.org/cmap/cgi-bin/cmap/viewer?data_source=sbt_cmap;ref_map_set_aid=GmComposite2003&ref_map_aids=GmComposite2003_L&comparative_map_left=GmConsensus40_L;highlight=%22Seed+Glu+1-5%22GmComposite2003_L) | 60.34 | 62.34 | F6 RIL | [Satt076](http://www.soybase.org/sbt/search/search_results.php?category=LocusName&search_term=Satt076) | N87-984-16 | TN93-99 | 101 |  |
| [Seed Glu 1-6](http://www.soybase.org/sbt/search/search_results.php?category=QTLName&search_term=Seed+Glu+1-6) | [M](http://www.soybase.org/cmap/cgi-bin/cmap/viewer?data_source=sbt_cmap;ref_map_set_aid=GmComposite2003&ref_map_aids=GmComposite2003_M&comparative_map_left=GmConsensus40_M;highlight=%22Seed+Glu+1-6%22GmComposite2003_M) | 32.47 | 34.47 | F6 RIL | [Satt567](http://www.soybase.org/sbt/search/search_results.php?category=LocusName&search_term=Satt567) | N87-984-16 | TN93-99 | 101 |  |
| [Seed Glu 1-7](http://www.soybase.org/sbt/search/search_results.php?category=QTLName&search_term=Seed+Glu+1-7) | [O](http://www.soybase.org/cmap/cgi-bin/cmap/viewer?data_source=sbt_cmap;ref_map_set_aid=GmComposite2003&ref_map_aids=GmComposite2003_O&comparative_map_left=GmConsensus40_O;highlight=%22Seed+Glu+1-7%22GmComposite2003_O) | 48.7 | 50.7 | F6 RIL | [Satt420](http://www.soybase.org/sbt/search/search_results.php?category=LocusName&search_term=Satt420) | N87-984-16 | TN93-99 | 101 |  |
| Seed His 1-1 |  |  |  | F6 RIL | [Satt380](http://www.soybase.org/sbt/search/search_results.php?category=LocusName&search_term=Satt380) | N87-984-16 | TN93-99 | 101 |  |
| [Seed Leu 1-1](http://www.soybase.org/sbt/search/search_results.php?category=QTLName&search_term=Seed+Leu+1-1) | [D1a](http://www.soybase.org/cmap/cgi-bin/cmap/viewer?data_source=sbt_cmap;ref_map_set_aid=GmComposite2003&ref_map_aids=GmComposite2003_D1a&comparative_map_left=GmConsensus40_D1a;highlight=%22Seed+Leu+1-1%22GmComposite2003_D1a) | 58 | 60 | F6 RIL | [Satt203](http://www.soybase.org/sbt/search/search_results.php?category=LocusName&search_term=Satt203) | N87-984-16 | TN93-99 | 101 |  |
| [Seed Leu 1-2](http://www.soybase.org/sbt/search/search_results.php?category=QTLName&search_term=Seed+Leu+1-2) | [D1b](http://www.soybase.org/cmap/cgi-bin/cmap/viewer?data_source=sbt_cmap;ref_map_set_aid=GmComposite2003&ref_map_aids=GmComposite2003_D1b&comparative_map_left=GmConsensus40_D1b;highlight=%22Seed+Leu+1-2%22GmComposite2003_D1b) | 115.34 | 117.34 | F6 RIL | [Satt274](http://www.soybase.org/sbt/search/search_results.php?category=LocusName&search_term=Satt274) | N87-984-16 | TN93-99 | 101 |  |
| [Seed Leu 1-3](http://www.soybase.org/sbt/search/search_results.php?category=QTLName&search_term=Seed+Leu+1-3) | [D2](http://www.soybase.org/cmap/cgi-bin/cmap/viewer?data_source=sbt_cmap;ref_map_set_aid=GmComposite2003&ref_map_aids=GmComposite2003_D2&comparative_map_left=GmConsensus40_D2;highlight=%22Seed+Leu+1-3%22GmComposite2003_D2) | 46.73 | 48.73 | F6 RIL | [Satt002](http://www.soybase.org/sbt/search/search_results.php?category=LocusName&search_term=Satt002) | N87-984-16 | TN93-99 | 101 |  |
| [Seed Leu 1-4](http://www.soybase.org/sbt/search/search_results.php?category=QTLName&search_term=Seed+Leu+1-4) | [G](http://www.soybase.org/cmap/cgi-bin/cmap/viewer?data_source=sbt_cmap;ref_map_set_aid=GmComposite2003&ref_map_aids=GmComposite2003_G&comparative_map_left=GmConsensus40_G;highlight=%22Seed+Leu+1-4%22GmComposite2003_G) | 11.74 | 13.74 | F6 RIL | [Satt570](http://www.soybase.org/sbt/search/search_results.php?category=LocusName&search_term=Satt570) | N87-984-16 | TN93-99 | 101 |  |
| [Seed Leu 1-5](http://www.soybase.org/sbt/search/search_results.php?category=QTLName&search_term=Seed+Leu+1-5) | [L](http://www.soybase.org/cmap/cgi-bin/cmap/viewer?data_source=sbt_cmap;ref_map_set_aid=GmComposite2003&ref_map_aids=GmComposite2003_L&comparative_map_left=GmConsensus40_L;highlight=%22Seed+Leu+1-5%22GmComposite2003_L) | 60.34 | 62.34 | F6 RIL | [Satt076](http://www.soybase.org/sbt/search/search_results.php?category=LocusName&search_term=Satt076) | N87-984-16 | TN93-99 | 101 |  |
| [Seed Leu 1-6](http://www.soybase.org/sbt/search/search_results.php?category=QTLName&search_term=Seed+Leu+1-6) | [M](http://www.soybase.org/cmap/cgi-bin/cmap/viewer?data_source=sbt_cmap;ref_map_set_aid=GmComposite2003&ref_map_aids=GmComposite2003_M&comparative_map_left=GmConsensus40_M;highlight=%22Seed+Leu+1-6%22GmComposite2003_M) | 32.47 | 34.47 | F6 RIL | [Satt567](http://www.soybase.org/sbt/search/search_results.php?category=LocusName&search_term=Satt567) | N87-984-16 | TN93-99 | 101 |  |
| [Seed Leu 1-7](http://www.soybase.org/sbt/search/search_results.php?category=QTLName&search_term=Seed+Leu+1-7) | [N](http://www.soybase.org/cmap/cgi-bin/cmap/viewer?data_source=sbt_cmap;ref_map_set_aid=GmComposite2003&ref_map_aids=GmComposite2003_N&comparative_map_left=GmConsensus40_N;highlight=%22Seed+Leu+1-7%22GmComposite2003_N) | 75.48 | 77.48 | F6 RIL | [Satt255](http://www.soybase.org/sbt/search/search_results.php?category=LocusName&search_term=Satt255) | N87-984-16 | TN93-99 | 101 |  |
| [Seed Met plus Cys 1-1](http://www.soybase.org/sbt/search/search_results.php?category=QTLName&search_term=Seed+Met+plus+Cys+1-1) | [D2](http://www.soybase.org/cmap/cgi-bin/cmap/viewer?data_source=sbt_cmap;ref_map_set_aid=GmComposite2003&ref_map_aids=GmComposite2003_D2&comparative_map_left=GmConsensus40_D2;highlight=%22Seed+Met+plus+Cys+1-1%22GmComposite2003_D2) | 46.73 | 48.73 | F6 RIL | [Satt002](http://www.soybase.org/sbt/search/search_results.php?category=LocusName&search_term=Satt002) | N87-984-16 | TN93-99 | 101 | Panthee et al. 2006A Quantitative Trait Loci controlling sulfur containing amino acids methionine and cysteine, in soybean seeds  Theor. Appl. Genet. 2006, 112(3):546-553 |
| [Seed Met plus Cys 1-2](http://www.soybase.org/sbt/search/search_results.php?category=QTLName&search_term=Seed+Met+plus+Cys+1-2) | [F](http://www.soybase.org/cmap/cgi-bin/cmap/viewer?data_source=sbt_cmap;ref_map_set_aid=GmComposite2003&ref_map_aids=GmComposite2003_F&comparative_map_left=GmConsensus40_F;highlight=%22Seed+Met+plus+Cys+1-2%22GmComposite2003_F) | 15.08 | 17.08 | F6 RIL | [Satt252](http://www.soybase.org/sbt/search/search_results.php?category=LocusName&search_term=Satt252) | N87-984-16 | TN93-99 | 101 |  |
| [Seed Met plus Cys 1-3](http://www.soybase.org/sbt/search/search_results.php?category=QTLName&search_term=Seed+Met+plus+Cys+1-3) | [M](http://www.soybase.org/cmap/cgi-bin/cmap/viewer?data_source=sbt_cmap;ref_map_set_aid=GmComposite2003&ref_map_aids=GmComposite2003_M&comparative_map_left=GmConsensus40_M;highlight=%22Seed+Met+plus+Cys+1-3%22GmComposite2003_M) | 6.84 | 8.84 | F6 RIL | [Satt590](http://www.soybase.org/sbt/search/search_results.php?category=LocusName&search_term=Satt590) | N87-984-16 | TN93-99 | 101 |  |
| [Seed Thr 1-1](http://www.soybase.org/sbt/search/search_results.php?category=QTLName&search_term=Seed+Thr+1-1) | [A1](http://www.soybase.org/cmap/cgi-bin/cmap/viewer?data_source=sbt_cmap;ref_map_set_aid=GmComposite2003&ref_map_aids=GmComposite2003_A1&comparative_map_left=GmConsensus40_A1;highlight=%22Seed+Thr+1-1%22GmComposite2003_A1) | 24.55 | 26.55 | F6 RIL | [Satt593](http://www.soybase.org/sbt/search/search_results.php?category=LocusName&search_term=Satt593) | N87-984-16 | TN93-99 | 101 | Panthee et al. 2006B Genomic regions associated with amino acid composition in soybean  Mol. Breed. 2006, 17(1):79-89 |
| [Seed Thr 1-2](http://www.soybase.org/sbt/search/search_results.php?category=QTLName&search_term=Seed+Thr+1-2) | [L](http://www.soybase.org/cmap/cgi-bin/cmap/viewer?data_source=sbt_cmap;ref_map_set_aid=GmComposite2003&ref_map_aids=GmComposite2003_L&comparative_map_left=GmConsensus40_L;highlight=%22Seed+Thr+1-2%22GmComposite2003_L) | 29.19 | 31.19 | F6 RIL | [Satt143](http://www.soybase.org/sbt/search/search_results.php?category=LocusName&search_term=Satt143) | N87-984-16 | TN93-99 | 101 |  |
| [Seed Thr 1-3](http://www.soybase.org/sbt/search/search_results.php?category=QTLName&search_term=Seed+Thr+1-3) | [D1b](http://www.soybase.org/cmap/cgi-bin/cmap/viewer?data_source=sbt_cmap;ref_map_set_aid=GmComposite2003&ref_map_aids=GmComposite2003_D1b&comparative_map_left=GmConsensus40_D1b;highlight=%22Seed+Thr+1-3%22GmComposite2003_D1b) | 115.34 | 117.34 | F6 RIL | [Satt274](http://www.soybase.org/sbt/search/search_results.php?category=LocusName&search_term=Satt274) | N87-984-16 | TN93-99 | 101 |  |
| [Seed Thr 1-4](http://www.soybase.org/sbt/search/search_results.php?category=QTLName&search_term=Seed+Thr+1-4) | [K](http://www.soybase.org/cmap/cgi-bin/cmap/viewer?data_source=sbt_cmap;ref_map_set_aid=GmComposite2003&ref_map_aids=GmComposite2003_K&comparative_map_left=GmConsensus40_K;highlight=%22Seed+Thr+1-4%22GmComposite2003_K) | 45.63 | 47.63 | F6 RIL | [Satt518](http://www.soybase.org/sbt/search/search_results.php?category=LocusName&search_term=Satt518) | N87-984-16 | TN93-99 | 101 |  |
| [Seed Thr 1-5](http://www.soybase.org/sbt/search/search_results.php?category=QTLName&search_term=Seed+Thr+1-5) | [L](http://www.soybase.org/cmap/cgi-bin/cmap/viewer?data_source=sbt_cmap;ref_map_set_aid=GmComposite2003&ref_map_aids=GmComposite2003_L&comparative_map_left=GmConsensus40_L;highlight=%22Seed+Thr+1-5%22GmComposite2003_L) | 0 | 2 | F6 RIL | [Satt495](http://www.soybase.org/sbt/search/search_results.php?category=LocusName&search_term=Satt495) | N87-984-16 | TN93-99 | 101 |  |
| [Seed Tyr 1-1](http://www.soybase.org/sbt/search/search_results.php?category=QTLName&search_term=Seed+Tyr+1-1) | [A2](http://www.soybase.org/cmap/cgi-bin/cmap/viewer?data_source=sbt_cmap;ref_map_set_aid=GmComposite2003&ref_map_aids=GmComposite2003_A2&comparative_map_left=GmConsensus40_A2;highlight=%22Seed+Tyr+1-1%22GmComposite2003_A2) | 124.37 | 126.37 | F6 RIL | [Satt133](http://www.soybase.org/sbt/search/search_results.php?category=LocusName&search_term=Satt133) | N87-984-16 | TN93-99 | 101 |  |
| [Seed Tyr 1-2](http://www.soybase.org/sbt/search/search_results.php?category=QTLName&search_term=Seed+Tyr+1-2) | [D1b](http://www.soybase.org/cmap/cgi-bin/cmap/viewer?data_source=sbt_cmap;ref_map_set_aid=GmComposite2003&ref_map_aids=GmComposite2003_D1b&comparative_map_left=GmConsensus40_D1b;highlight=%22Seed+Tyr+1-2%22GmComposite2003_D1b) | 115.34 | 117.34 | F6 RIL | [Satt274](http://www.soybase.org/sbt/search/search_results.php?category=LocusName&search_term=Satt274) | N87-984-16 | TN93-99 | 101 |  |
| [Seed Tyr 1-3](http://www.soybase.org/sbt/search/search_results.php?category=QTLName&search_term=Seed+Tyr+1-3) | [E](http://www.soybase.org/cmap/cgi-bin/cmap/viewer?data_source=sbt_cmap;ref_map_set_aid=GmComposite2003&ref_map_aids=GmComposite2003_E&comparative_map_left=GmConsensus40_E;highlight=%22Seed+Tyr+1-3%22GmComposite2003_E) | 43.75 | 45.75 | F6 RIL | [Satt185](http://www.soybase.org/sbt/search/search_results.php?category=LocusName&search_term=Satt185) | N87-984-16 | TN93-99 | 101 |  |
| [Seed Tyr 1-4](http://www.soybase.org/sbt/search/search_results.php?category=QTLName&search_term=Seed+Tyr+1-4) | [K](http://www.soybase.org/cmap/cgi-bin/cmap/viewer?data_source=sbt_cmap;ref_map_set_aid=GmComposite2003&ref_map_aids=GmComposite2003_K&comparative_map_left=GmConsensus40_K;highlight=%22Seed+Tyr+1-4%22GmComposite2003_K) | 29.28 | 31.28 | F6 RIL | [Satt102](http://www.soybase.org/sbt/search/search_results.php?category=LocusName&search_term=Satt102) | N87-984-16 | TN93-99 | 101 |  |
| [Seed Tyr 1-5](http://www.soybase.org/sbt/search/search_results.php?category=QTLName&search_term=Seed+Tyr+1-5) | [L](http://www.soybase.org/cmap/cgi-bin/cmap/viewer?data_source=sbt_cmap;ref_map_set_aid=GmComposite2003&ref_map_aids=GmComposite2003_L&comparative_map_left=GmConsensus40_L;highlight=%22Seed+Tyr+1-5%22GmComposite2003_L) | 33.54 | 35.54 | F6 RIL | [Satt313](http://www.soybase.org/sbt/search/search_results.php?category=LocusName&search_term=Satt313) | N87-984-16 | TN93-99 | 101 |  |
| Seed Arg 1-1 |  |  |  | F6 RIL | [Satt274](http://www.soybase.org/sbt/search/search_results.php?category=LocusName&search_term=Satt274) | N87-984-16 | TN93-99 | 101 |  |
| [Seed Cys 1-1](http://www.soybase.org/sbt/search/search_results.php?category=QTLName&search_term=Seed+Cys+1-1) | [F](http://www.soybase.org/cmap/cgi-bin/cmap/viewer?data_source=sbt_cmap;ref_map_set_aid=GmComposite2003&ref_map_aids=GmComposite2003_F&comparative_map_left=GmConsensus40_F;highlight=%22Seed+Cys+1-1%22GmComposite2003_F) | 15.08 | 17.08 | F6 RIL | [Satt252](http://www.soybase.org/sbt/search/search_results.php?category=LocusName&search_term=Satt252) | N87-984-16 | TN93-99 | 101 |  |
| [Seed Cys 1-2](http://www.soybase.org/sbt/search/search_results.php?category=QTLName&search_term=Seed+Cys+1-2) | [G](http://www.soybase.org/cmap/cgi-bin/cmap/viewer?data_source=sbt_cmap;ref_map_set_aid=GmComposite2003&ref_map_aids=GmComposite2003_G&comparative_map_left=GmConsensus40_G;highlight=%22Seed+Cys+1-2%22GmComposite2003_G) | 20.88 | 22.88 | F6 RIL | [Satt235](http://www.soybase.org/sbt/search/search_results.php?category=LocusName&search_term=Satt235) | N87-984-16 | TN93-99 | 101 |  |
| [Seed Gly 1-1](http://www.soybase.org/sbt/search/search_results.php?category=QTLName&search_term=Seed+Gly+1-1) | [A1](http://www.soybase.org/cmap/cgi-bin/cmap/viewer?data_source=sbt_cmap;ref_map_set_aid=GmComposite2003&ref_map_aids=GmComposite2003_A1&comparative_map_left=GmConsensus40_A1;highlight=%22Seed+Gly+1-1%22GmComposite2003_A1) | 24.55 | 26.55 | F6 RIL | [Satt593](http://www.soybase.org/sbt/search/search_results.php?category=LocusName&search_term=Satt593) | N87-984-16 | TN93-99 | 101 |  |
| [Seed Gly 1-2](http://www.soybase.org/sbt/search/search_results.php?category=QTLName&search_term=Seed+Gly+1-2) | [K](http://www.soybase.org/cmap/cgi-bin/cmap/viewer?data_source=sbt_cmap;ref_map_set_aid=GmComposite2003&ref_map_aids=GmComposite2003_K&comparative_map_left=GmConsensus40_K;highlight=%22Seed+Gly+1-2%22GmComposite2003_K) | 45.63 | 47.63 | F6 RIL | [Satt518](http://www.soybase.org/sbt/search/search_results.php?category=LocusName&search_term=Satt518) | N87-984-16 | TN93-99 | 101 |  |
| [Seed Gly 1-3](http://www.soybase.org/sbt/search/search_results.php?category=QTLName&search_term=Seed+Gly+1-3) | [D1b](http://www.soybase.org/cmap/cgi-bin/cmap/viewer?data_source=sbt_cmap;ref_map_set_aid=GmComposite2003&ref_map_aids=GmComposite2003_D1b&comparative_map_left=GmConsensus40_D1b;highlight=%22Seed+Gly+1-3%22GmComposite2003_D1b) | 115.34 | 117.34 | F6 RIL | [Satt274](http://www.soybase.org/sbt/search/search_results.php?category=LocusName&search_term=Satt274) | N87-984-16 | TN93-99 | 101 |  |
| [Seed Gly 1-4](http://www.soybase.org/sbt/search/search_results.php?category=QTLName&search_term=Seed+Gly+1-4) | [L](http://www.soybase.org/cmap/cgi-bin/cmap/viewer?data_source=sbt_cmap;ref_map_set_aid=GmComposite2003&ref_map_aids=GmComposite2003_L&comparative_map_left=GmConsensus40_L;highlight=%22Seed+Gly+1-4%22GmComposite2003_L) | 0 | 2 | F6 RIL | [Satt495](http://www.soybase.org/sbt/search/search_results.php?category=LocusName&search_term=Satt495) | N87-984-16 | TN93-99 | 101 |  |
| [Seed Gly 1-5](http://www.soybase.org/sbt/search/search_results.php?category=QTLName&search_term=Seed+Gly+1-5) | [L](http://www.soybase.org/cmap/cgi-bin/cmap/viewer?data_source=sbt_cmap;ref_map_set_aid=GmComposite2003&ref_map_aids=GmComposite2003_L&comparative_map_left=GmConsensus40_L;highlight=%22Seed+Gly+1-5%22GmComposite2003_L) | 29.19 | 31.19 | F6 RIL | [Satt143](http://www.soybase.org/sbt/search/search_results.php?category=LocusName&search_term=Satt143) | N87-984-16 | TN93-99 | 101 |  |
| [Seed Ile 1-1](http://www.soybase.org/sbt/search/search_results.php?category=QTLName&search_term=Seed+Ile+1-1) | [A2](http://www.soybase.org/cmap/cgi-bin/cmap/viewer?data_source=sbt_cmap;ref_map_set_aid=GmComposite2003&ref_map_aids=GmComposite2003_A2&comparative_map_left=GmConsensus40_A2;highlight=%22Seed+Ile+1-1%22GmComposite2003_A2) | 35.77 | 37.77 | F6 RIL | Satt177 | N87-984-16 | TN93-99 | 101 |  |
| [Seed Ile 1-2](http://www.soybase.org/sbt/search/search_results.php?category=QTLName&search_term=Seed+Ile+1-2) | [B2](http://www.soybase.org/cmap/cgi-bin/cmap/viewer?data_source=sbt_cmap;ref_map_set_aid=GmComposite2003&ref_map_aids=GmComposite2003_B2&comparative_map_left=GmConsensus40_B2;highlight=%22Seed+Ile+1-2%22GmComposite2003_B2) | 54.2 | 56.2 | F6 RIL | Satt168 | N87-984-16 | TN93-99 | 101 |  |
| [Seed Ile 1-3](http://www.soybase.org/sbt/search/search_results.php?category=QTLName&search_term=Seed+Ile+1-3) | [D1a](http://www.soybase.org/cmap/cgi-bin/cmap/viewer?data_source=sbt_cmap;ref_map_set_aid=GmComposite2003&ref_map_aids=GmComposite2003_D1a&comparative_map_left=GmConsensus40_D1a;highlight=%22Seed+Ile+1-3%22GmComposite2003_D1a) | 58 | 60 | F6 RIL | Satt203 | N87-984-16 | TN93-99 | 101 |  |
| [Seed Ile 1-4](http://www.soybase.org/sbt/search/search_results.php?category=QTLName&search_term=Seed+Ile+1-4) | [F](http://www.soybase.org/cmap/cgi-bin/cmap/viewer?data_source=sbt_cmap;ref_map_set_aid=GmComposite2003&ref_map_aids=GmComposite2003_F&comparative_map_left=GmConsensus40_F;highlight=%22Seed+Ile+1-4%22GmComposite2003_F) | 15.08 | 17.08 | F6 RIL | Satt252 | N87-984-16 | TN93-99 | 101 |  |
| [Seed Ile 1-5](http://www.soybase.org/sbt/search/search_results.php?category=QTLName&search_term=Seed+Ile+1-5) | [L](http://www.soybase.org/cmap/cgi-bin/cmap/viewer?data_source=sbt_cmap;ref_map_set_aid=GmComposite2003&ref_map_aids=GmComposite2003_L&comparative_map_left=GmConsensus40_L;highlight=%22Seed+Ile+1-5%22GmComposite2003_L) | 33.54 | 35.54 | F6 RIL | Satt313 | N87-984-16 | TN93-99 | 101 |  |
| [Seed Lys 1-1](http://www.soybase.org/sbt/search/search_results.php?category=QTLName&search_term=Seed+Lys+1-1) | [D1a](http://www.soybase.org/cmap/cgi-bin/cmap/viewer?data_source=sbt_cmap;ref_map_set_aid=GmComposite2003&ref_map_aids=GmComposite2003_D1a&comparative_map_left=GmConsensus40_D1a;highlight=%22Seed+Lys+1-1%22GmComposite2003_D1a) | 16.52 | 18.52 | F6 RIL | Satt184 | N87-984-16 | TN93-99 | 101 |  |
| [Seed Lys 1-2](http://www.soybase.org/sbt/search/search_results.php?category=QTLName&search_term=Seed+Lys+1-2) | [E](http://www.soybase.org/cmap/cgi-bin/cmap/viewer?data_source=sbt_cmap;ref_map_set_aid=GmComposite2003&ref_map_aids=GmComposite2003_E&comparative_map_left=GmConsensus40_E;highlight=%22Seed+Lys+1-2%22GmComposite2003_E) | 43.75 | 44.75 | F6 RIL | Satt185 | N87-984-16 | TN93-99 | 101 |  |
| [Seed Lys 1-3](http://www.soybase.org/sbt/search/search_results.php?category=QTLName&search_term=Seed+Lys+1-3) | [G](http://www.soybase.org/cmap/cgi-bin/cmap/viewer?data_source=sbt_cmap;ref_map_set_aid=GmComposite2003&ref_map_aids=GmComposite2003_G&comparative_map_left=GmConsensus40_G;highlight=%22Seed+Lys+1-3%22GmComposite2003_G) | 50.68 | 52.68 | F6 RIL | Satt427 | N87-984-16 | TN93-99 | 101 |  |
| [Seed Met 1-1](http://www.soybase.org/sbt/search/search_results.php?category=QTLName&search_term=Seed+Met+1-1) | [F](http://www.soybase.org/cmap/cgi-bin/cmap/viewer?data_source=sbt_cmap;ref_map_set_aid=GmComposite2003&ref_map_aids=GmComposite2003_F&comparative_map_left=GmConsensus40_F;highlight=%22Seed+Met+1-1%22GmComposite2003_F) | 15.08 | 17.08 | F6 RIL | Satt252 | N87-984-16 | TN93-99 | 101 |  |
| [Seed Met 1-2](http://www.soybase.org/sbt/search/search_results.php?category=QTLName&search_term=Seed+Met+1-2) | [G](http://www.soybase.org/cmap/cgi-bin/cmap/viewer?data_source=sbt_cmap;ref_map_set_aid=GmComposite2003&ref_map_aids=GmComposite2003_G&comparative_map_left=GmConsensus40_G;highlight=%22Seed+Met+1-2%22GmComposite2003_G) | 56.32 | 58.32 | F6 RIL | Satt564 | N87-984-16 | TN93-99 | 101 |  |
| [Seed Met 1-3](http://www.soybase.org/sbt/search/search_results.php?category=QTLName&search_term=Seed+Met+1-3) | [M](http://www.soybase.org/cmap/cgi-bin/cmap/viewer?data_source=sbt_cmap;ref_map_set_aid=GmComposite2003&ref_map_aids=GmComposite2003_M&comparative_map_left=GmConsensus40_M;highlight=%22Seed+Met+1-3%22GmComposite2003_M) | 6.84 | 8.84 | F6 RIL | Satt590 | N87-984-16 | TN93-99 | 101 |  |
| [Seed Met plus Cys 1-1](http://www.soybase.org/sbt/search/search_results.php?category=QTLName&search_term=Seed+Met+plus+Cys+1-1) | [D2](http://www.soybase.org/cmap/cgi-bin/cmap/viewer?data_source=sbt_cmap;ref_map_set_aid=GmComposite2003&ref_map_aids=GmComposite2003_D2&comparative_map_left=GmConsensus40_D2;highlight=%22Seed+Met+plus+Cys+1-1%22GmComposite2003_D2) | 46.73 | 48.73 | F6 RIL | Satt002 | N87-984-16 | TN93-99 | 101 | Panthee et al. 2006A Quantitative Trait Loci controlling sulfur containing amino acids methionine and cysteine, in soybean seeds  Theor. Appl. Genet. 2006, 112(3):546-553 |
| [Seed Met plus Cys 1-2](http://www.soybase.org/sbt/search/search_results.php?category=QTLName&search_term=Seed+Met+plus+Cys+1-2) | [F](http://www.soybase.org/cmap/cgi-bin/cmap/viewer?data_source=sbt_cmap;ref_map_set_aid=GmComposite2003&ref_map_aids=GmComposite2003_F&comparative_map_left=GmConsensus40_F;highlight=%22Seed+Met+plus+Cys+1-2%22GmComposite2003_F) | 15.08 | 17.08 | F6 RIL | Satt252 | N87-984-16 | TN93-99 | 101 |  |
| [Seed Met plus Cys 1-3](http://www.soybase.org/sbt/search/search_results.php?category=QTLName&search_term=Seed+Met+plus+Cys+1-3) | [M](http://www.soybase.org/cmap/cgi-bin/cmap/viewer?data_source=sbt_cmap;ref_map_set_aid=GmComposite2003&ref_map_aids=GmComposite2003_M&comparative_map_left=GmConsensus40_M;highlight=%22Seed+Met+plus+Cys+1-3%22GmComposite2003_M) | 6.84 | 8.84 | F6 RIL | Satt590 | N87-984-16 | TN93-99 | 101 |  |
| [Seed Ser 1-1](http://www.soybase.org/sbt/search/search_results.php?category=QTLName&search_term=Seed+Ser+1-1) | [A2](http://www.soybase.org/cmap/cgi-bin/cmap/viewer?data_source=sbt_cmap;ref_map_set_aid=GmComposite2003&ref_map_aids=GmComposite2003_A2&comparative_map_left=GmConsensus40_A2;highlight=%22Seed+Ser+1-1%22GmComposite2003_A2) | 106.05 | 108.05 | F6 RIL | Satt437 | N87-984-16 | TN93-99 | 101 | Panthee et al. 2006B Genomic regions associated with amino acid composition in soybean  Mol. Breed. 2006, 17(1):79-89 |
| [Seed Ser 1-2](http://www.soybase.org/sbt/search/search_results.php?category=QTLName&search_term=Seed+Ser+1-2) | [C1](http://www.soybase.org/cmap/cgi-bin/cmap/viewer?data_source=sbt_cmap;ref_map_set_aid=GmComposite2003&ref_map_aids=GmComposite2003_C1&comparative_map_left=GmConsensus40_C1;highlight=%22Seed+Ser+1-2%22GmComposite2003_C1) | 73.45 | 75.45 | F6 RIL | Satt139 | N87-984-16 | TN93-99 | 101 |  |
| [Seed Ser 1-3](http://www.soybase.org/sbt/search/search_results.php?category=QTLName&search_term=Seed+Ser+1-3) | [K](http://www.soybase.org/cmap/cgi-bin/cmap/viewer?data_source=sbt_cmap;ref_map_set_aid=GmComposite2003&ref_map_aids=GmComposite2003_K&comparative_map_left=GmConsensus40_K;highlight=%22Seed+Ser+1-3%22GmComposite2003_K) | 29.28 | 31.28 | F6 RIL | Satt102 | N87-984-16 | TN93-99 | 101 |  |
| [Seed Ser 1-4](http://www.soybase.org/sbt/search/search_results.php?category=QTLName&search_term=Seed+Ser+1-4) | [L](http://www.soybase.org/cmap/cgi-bin/cmap/viewer?data_source=sbt_cmap;ref_map_set_aid=GmComposite2003&ref_map_aids=GmComposite2003_L&comparative_map_left=GmConsensus40_L;highlight=%22Seed+Ser+1-4%22GmComposite2003_L) | 0 | 2 | F6 RIL | Satt495 | N87-984-16 | TN93-99 | 101 |  |
| [Seed Ser 1-5](http://www.soybase.org/sbt/search/search_results.php?category=QTLName&search_term=Seed+Ser+1-5) | [L](http://www.soybase.org/cmap/cgi-bin/cmap/viewer?data_source=sbt_cmap;ref_map_set_aid=GmComposite2003&ref_map_aids=GmComposite2003_L&comparative_map_left=GmConsensus40_L;highlight=%22Seed+Ser+1-5%22GmComposite2003_L) | 29.19 | 31.19 | F6 RIL | Satt143 | N87-984-16 | TN93-99 | 101 |  |
| [Seed Ser 1-6](http://www.soybase.org/sbt/search/search_results.php?category=QTLName&search_term=Seed+Ser+1-6) | [M](http://www.soybase.org/cmap/cgi-bin/cmap/viewer?data_source=sbt_cmap;ref_map_set_aid=GmComposite2003&ref_map_aids=GmComposite2003_M&comparative_map_left=GmConsensus40_M;highlight=%22Seed+Ser+1-6%22GmComposite2003_M) | 32.47 | 34.47 | F6 RIL | Satt567 | N87-984-16 | TN93-99 | 101 |  |
| [Seed Trp 1-1](http://www.soybase.org/sbt/search/search_results.php?category=QTLName&search_term=Seed+Trp+1-1) | [A1](http://www.soybase.org/cmap/cgi-bin/cmap/viewer?data_source=sbt_cmap;ref_map_set_aid=GmComposite2003&ref_map_aids=GmComposite2003_A1&comparative_map_left=GmConsensus40_A1;highlight=%22Seed+Trp+1-1%22GmComposite2003_A1) | 92.23 | 94.23 | F6 RIL | Satt236 | N87-984-16 | TN93-99 | 101 |  |
| [Seed Trp 1-2](http://www.soybase.org/sbt/search/search_results.php?category=QTLName&search_term=Seed+Trp+1-2) | [C1](http://www.soybase.org/cmap/cgi-bin/cmap/viewer?data_source=sbt_cmap;ref_map_set_aid=GmComposite2003&ref_map_aids=GmComposite2003_C1&comparative_map_left=GmConsensus40_C1;highlight=%22Seed+Trp+1-2%22GmComposite2003_C1) | 73.45 | 75.45 | F6 RIL | Satt139 | N87-984-16 | TN93-99 | 101 |  |
| [Seed Trp 1-3](http://www.soybase.org/sbt/search/search_results.php?category=QTLName&search_term=Seed+Trp+1-3) | [D1a](http://www.soybase.org/cmap/cgi-bin/cmap/viewer?data_source=sbt_cmap;ref_map_set_aid=GmComposite2003&ref_map_aids=GmComposite2003_D1a&comparative_map_left=GmConsensus40_D1a;highlight=%22Seed+Trp+1-3%22GmComposite2003_D1a) | 58 | 60 | F6 RIL | Satt203 | N87-984-16 | TN93-99 | 101 |  |
| [Seed Trp 1-4](http://www.soybase.org/sbt/search/search_results.php?category=QTLName&search_term=Seed+Trp+1-4) | [D1b](http://www.soybase.org/cmap/cgi-bin/cmap/viewer?data_source=sbt_cmap;ref_map_set_aid=GmComposite2003&ref_map_aids=GmComposite2003_D1b&comparative_map_left=GmConsensus40_D1b;highlight=%22Seed+Trp+1-4%22GmComposite2003_D1b) | 74.66 | 76.66 | F6 RIL | Satt537 | N87-984-16 | TN93-99 | 101 |  |
| [Seed Trp 1-5](http://www.soybase.org/sbt/search/search_results.php?category=QTLName&search_term=Seed+Trp+1-5) | [G](http://www.soybase.org/cmap/cgi-bin/cmap/viewer?data_source=sbt_cmap;ref_map_set_aid=GmComposite2003&ref_map_aids=GmComposite2003_G&comparative_map_left=GmConsensus40_G;highlight=%22Seed+Trp+1-5%22GmComposite2003_G) | 11.74 | 13.74 | F6 RIL | Satt570 | N87-984-16 | TN93-99 | 101 |  |
| [Seed Trp 1-6](http://www.soybase.org/sbt/search/search_results.php?category=QTLName&search_term=Seed+Trp+1-6) | [I](http://www.soybase.org/cmap/cgi-bin/cmap/viewer?data_source=sbt_cmap;ref_map_set_aid=GmComposite2003&ref_map_aids=GmComposite2003_I&comparative_map_left=GmConsensus40_I;highlight=%22Seed+Trp+1-6%22GmComposite2003_I) | 81.77 | 83.77 | F6 RIL | Satt292 | N87-984-16 | TN93-99 | 101 |  |
| [Seed Val 1-1](http://www.soybase.org/sbt/search/search_results.php?category=QTLName&search_term=Seed+Val+1-1) | [B2](http://www.soybase.org/cmap/cgi-bin/cmap/viewer?data_source=sbt_cmap;ref_map_set_aid=GmComposite2003&ref_map_aids=GmComposite2003_B2&comparative_map_left=GmConsensus40_B2;highlight=%22Seed+Val+1-1%22GmComposite2003_B2) | 54.2 | 56.2 | F6 RIL | Satt168 | N87-984-16 | TN93-99 | 101 |  |
| [Seed Val 1-2](http://www.soybase.org/sbt/search/search_results.php?category=QTLName&search_term=Seed+Val+1-2) | [D1b](http://www.soybase.org/cmap/cgi-bin/cmap/viewer?data_source=sbt_cmap;ref_map_set_aid=GmComposite2003&ref_map_aids=GmComposite2003_D1b&comparative_map_left=GmConsensus40_D1b;highlight=%22Seed+Val+1-2%22GmComposite2003_D1b) | 74.66 | 76.66 | F6 RIL | Satt537 | N87-984-16 | TN93-99 | 101 |  |
| [Seed Val 1-3](http://www.soybase.org/sbt/search/search_results.php?category=QTLName&search_term=Seed+Val+1-3) | [F](http://www.soybase.org/cmap/cgi-bin/cmap/viewer?data_source=sbt_cmap;ref_map_set_aid=GmComposite2003&ref_map_aids=GmComposite2003_F&comparative_map_left=GmConsensus40_F;highlight=%22Seed+Val+1-3%22GmComposite2003_F) | 15.08 | 17.08 | F6 RIL | Satt252 | N87-984-16 | TN93-99 | 101 |  |
| [Seed Val 1-4](http://www.soybase.org/sbt/search/search_results.php?category=QTLName&search_term=Seed+Val+1-4) | [L](http://www.soybase.org/cmap/cgi-bin/cmap/viewer?data_source=sbt_cmap;ref_map_set_aid=GmComposite2003&ref_map_aids=GmComposite2003_L&comparative_map_left=GmConsensus40_L;highlight=%22Seed+Val+1-4%22GmComposite2003_L) | 29.19 | 31.19 | F6 RIL | Satt143 | N87-984-16 | TN93-99 | 101 |  |
| qLys_Gm08 | A2 | 31.7 | 35.6 | F5 | BARC-055265–Satt089 | Benning | Danbaekkong | 140 | Warrington, C., et al. (2015) QTL for seed protein and amino acids in the Benning× Danbaekkong soybean population. Theor. Appl. Genet. 128, 839-850 |
| qLys_Gm20 | I | 14.7 | 20.6 | F5 | GSM0012-Satt354 | Benning | Danbaekkong | 141 |  |
| qThr_Gm01 | D1a | 53.1 | 64.4 | F5 | BARC-035219–BARC-018211 | Benning | Danbaekkong | 142 |  |
| qThr_Gm09 | K | 30.8 | 32 | F5 | BARC-31967–BARC-042449 | Benning | Danbaekkong | 143 |  |
| qThr_Gm17 | D2 | 51.8 | 61 | F5 | Satt256-BARC–039151 | Benning | Danbaekkong | 144 |  |
| qThr_Gm20 | I | 14.6 | 16.1 | F5 | GSM0012–BARC-020713 | Benning | Danbaekkong | 145 |  |
| qMet_Gm06 | C2 | 28.2 | 33 | F5 | BARC-055889–BARC-048217 | Benning | Danbaekkong | 146 |  |
| qMet_Gm09 | K | 30.8 | 32 | F5 | BARC-31967–BARC-042449 | Benning | Danbaekkong | 147 |  |
| qMet_Gm10 | O | 52.9 | 57.8 | F5 | Satt592–BARC_043247 | Benning | Danbaekkong | 148 |  |
| qMet_Gm20 | I | 14.6 | 17 | F5 | GSM0012–BARC-020713 | Benning | Danbaekkong | 149 |  |
| qCys_Gm10 | O | 52.7 | 57.8 | F5 | Satt592–BARC_043247 | Benning | Danbaekkong | 150 |  |
| qMet + Cys_Gm10 | O | 52 | 57.8 | F5 | Satt592–BARC_043247 | Benning | Danbaekkong | 151 |  |
| qMet + Cys_Gm20 | I | 14.6 | 19.5 | F5 | GSM0012–BARC-020713 | Benning | Danbaekkong | 152 |  |
