# Supplementary material for: Molecular mapping and genomics of soybean seed protein: a review and perspective for the future
Source: Theor Appl Genet. 2017 Aug 11;130(10):1975–91. doi: 10.1007/s00122-017-2955-8 (PMC5606949; doi:10.1007/s00122-017-2955-8)
Supplement: Supplementary file 4 — Supplementary material 4 (DOCX 20 kb) [file 122_2017_2955_MOESM4_ESM.docx]

**Supplementary Table 4:** List of duplicated QTL regions for Chr. 20 and Chr. 15 identified using synteny analysis.

| Chr.20 (Glyma1.01 id) | Chr.20 (Wm82a2.V1.0 ID) | Chr. 10 (Wm82a2.V1.0) | Putative Function | Pfam/Arabidopsis Homologs |
| --- | --- | --- | --- | --- |
| Glyma20g20950 | Glyma.20G082000 | Glyma.10G132200 | Auxin-responsive protein n=1 Tax=Brassica rapa subsp. pekinensis RepID=M4EUM1_BRARP | NA |
| Glyma20g20980 | Glyma.20G082300 | - | MYB transcription factor n=1 Tax=Medicago truncatula RepID=G7I6P8_MEDTR | Myb-like DNA-binding domain |
| Glyma20g21047 | Glyma.20G082600 | - | Auxin-induced protein 5NG4 n=2 Tax=Medicago truncatula RepID=G7K2S5_MEDTR | NA |
| Glyma20g21064 | Glyma.20G082700 | - | bidirectional sugar transporter SWEET17 n=1 Tax=Arabidopsis thaliana RepID=UPI0001A7B0D3 | Sugar efflux transporter for intercellular exchange |
| Glyma20g21100 | Glyma.20G082800 | Glyma.10G134100 | Chloroplast RNA binding protein n=1 Tax=Phaseolus vulgaris RepID=Q41124_PHAVU | RNA recognition motif. (a.k.a. RRM, RBD, or RNP domain) |
| Glyma20g21115 | Glyma.20G082900 | - | Ribosomal protein-like protein n=1 Tax=Medicago truncatula RepID=G7L5H5_MEDTR | Plant transposon protein |
| Glyma20g21130 | Glyma.20G083000 | Glyma.10G133900 | Cysteine-rich repeat secretory protein n=1 Tax=Medicago truncatula RepID=G7I3U3_MEDTR | Salt stress response/antifungal |
| Glyma20g21151 | Glyma.20G083100 | - | Amino acid transporter n=1 Tax=Medicago truncatula RepID=G7K7B7_MEDTR | NA |
| Glyma20g21171 | Glyma.20G083200 | Glyma.10G132700 | HAT family dimerization domain containing protein n=1 Tax=Medicago truncatula RepID=G7JBV6_MEDTR | hAT family C-terminal dimerisation region |
| Glyma20g21190 | Glyma.20G083300 | Glyma.10G133100 | 40S ribosomal protein S3-3-like protein n=1 Tax=Phaseolus vulgaris RepID=T2DLX0_PHAVU | KH domain; "Ribosomal protein S3, C-terminal domain" |
| Glyma20g21200 | Glyma.20G083500 | Glyma.10G133200 | Syntaxin family protein n=1 Tax=Medicago truncatula RepID=G7JVG6_MEDTR | Syntaxin 6, N-terminal |
| Glyma20g21211 | Glyma.20G083600 | Glyma.10G133300 | NA | NA |
| Glyma20g21220 | Glyma.20G083700 | Glyma.10G133400 | NA | NA |
| Glyma20g21230 | Glyma.20G083800 | Glyma.10G133500 | Histone H4 n=1 Tax=Glycine max RepID=K7M9J2_SOYBN | Core histone H2A/H2B/H3/H4 |
| Glyma20g21250 | Glyma.20G084000 | - | Small nuclear ribonucleoprotein F n=1 Tax=Medicago truncatula RepID=G7JVF0_MEDTR | LSM domain |
| Glyma20g21270 | Glyma.20G084100 | Glyma.10G132800 | Calcium-binding EF hand family protein n=1 Tax=Arabidopsis lyrata subsp. lyrata RepID=D7KEB8_ARALL | Tetratricopeptide repeat |
| Glyma20g21301 | Glyma.20G084200 | - | NAD(P)H-quinone oxidoreductase subunit H, chloroplastic n=1 Tax=Glycine stenophita RepID=R9ZQR3_9FABA | Respiratory-chain NADH dehydrogenase, 49 Kd subunit |
| Glyma20g21330 | Glyma.20G084500 | Glyma.10G133700 | Pre-mRNA-processing factor-like protein n=1 Tax=Medicago truncatula RepID=G7I632_MEDTR | Prp19/Pso4-like; "WD domain, G-beta repeat" |
| Glyma20g21351 | Glyma.20G084900 | - | HAT family dimerization domain containing protein n=1 Tax=Medicago truncatula RepID=G7LIC7_MEDTR | BED zinc finger; "hAT family C-terminal dimerisation region"; "Protein of unknown function (DUF 659)" |
| Glyma20g21361 | Glyma.20G085000 | - | Conserved oligomeric Golgi complex subunit n=1 Tax=Medicago truncatula RepID=G7K400_MEDTR | Conserved oligomeric complex COG6 |
| Glyma20g21376 | Glyma.20G085100 | Glyma.10G134400 | Zinc finger-like protein n=1 Tax=Cucumis sativus RepID=B0F827_CUCSA | CCT motif |
| Glyma20g21383 | Glyma.20G085200 | - | LIM transcription factor n=2 Tax=Eucalyptus RepID=I0IK03_9MYRT | LIM domain |
| Glyma20g21420 | Glyma.20G085300 | Glyma.10G134500 | F19P19.4 protein n=1 Tax=Arabidopsis thaliana RepID=P93811_ARATH | Domain of unknown function (DUF3527) |
| Glyma20g21431 | Glyma.20G085400 | - | 60S ribosomal protein L23 n=1 Tax=Nicotiana tabacum RepID=RL23_TOBAC | Ribosomal protein L14p/L23e |
| Glyma20g21440 | Glyma.20G085500 | - | Stem 28 kDa glycoprotein n=1 Tax=Medicago truncatula RepID=G7I6M3_MEDTR | HAD superfamily, subfamily IIIB (Acid phosphatase) |
| Glyma20g21466 | Glyma.20G085700 | - | NA | NA |
| Glyma20g21493 | Glyma.20G085800 | Glyma.10G136100 | Eukaryotic translation initiation factor iso4E n=2 Tax=Phaseolus vulgaris RepID=D3TI64_PHAVU | Eukaryotic initiation factor 4E |
| Glyma20g21520 | Glyma.20G085900 | Glyma.10G136300 | Protein PROTON GRADIENT REGULATION n=1 Tax=Medicago truncatula RepID=G7I6M5_MEDTR | NA |
| Glyma20g21535 | Glyma.20G086000 | - | NA | Family of unknown function (DUF716) |
| Glyma20g21550 | Glyma.20G086100 | Glyma.10G136400 | OSJNBa0004N05.15 protein n=4 Tax=Oryza sativa RepID=Q7XPZ3_ORYSJ | Arp2/3 complex, 34 kD subunit p34-Arc |
| Glyma20g21600 | Glyma.20G086200 | Glyma.10G136600 | NA | NA |
| Glyma20g21610 | Glyma.20G086400 | Glyma.10G136800 | protein n=1 Tax=Arabidopsis thaliana RepID=P93812_ARATH | Protein of unknown function (DUF810) |
| Glyma20g21660 | Glyma.20G086700 | - | Ubiquitin carboxyl-terminal hydrolase n=1 Tax=Medicago truncatula RepID=G7I611_MEDTR | MATH domain |
| Glyma20g21693 | Glyma.20G086800 | - | Cucumisin n=1 Tax=Siraitia grosvenorii RepID=K7NBW1_SIRGR | PA domain |
| Glyma20g21726 | Glyma.20G086900 | - | Delta-1-pyrroline-5-carboxylate dehydrogenase 1 protein n=2 Tax=Medicago RepID=G7JPY4_MEDTR | Aldehyde dehydrogenase family |
| Glyma20g21780 | Glyma.20G087000 | - | Ethylene receptor family protein n=2 Tax=Populus trichocarpa RepID=B9HKG1_POPTR | GAF domain; "His Kinase A (phospho-acceptor) domain"; "Response regulator receiver domain" |
| Glyma20g21810 | Glyma.20G087200 | - | DNA-binding protein n=2 Tax=Coffea RepID=F2Y9E5_COFAR | Domain of unknown function (DUF296) |

| Chr.15 (Glyma1.01 id) | Chr.15 (Wm82a2.V1.0 ID) | Chr. 8 (Wm82a2.V1.0) | Putative Function | Pfam/Arabidopsis Homologs |
| --- | --- | --- | --- | --- |
| Glyma15g05400 | Glyma.15G048600 | - | UniRef100_G7IL66 COP9 signalosome complex subunit n=1 Tax=Medicago truncatula RepID=G7IL66_MEDTR | AT4G14110.1\|Symbols:COP9, CSN8, FUS7, EMB143\|COP9 signalosome, subunit CSN8\|chr4:8133049-8134867 REVERSE LENGTH=197 |
| Glyma15g05410 | Glyma.15G048700 | Glyma.08G184100 | UniRef100_A2Q483 Pinin/SDK/memA protein n=1 Tax=Medicago truncatula RepID=A2Q483_MEDTR | AT1G15200.1\|Symbols:\|protein-protein interaction regulator family protein\|chr1:5228477-5231017 REVERSE LENGTH=423 |
| Glyma15g05420 | Glyma.15G048800 | - | UniRef100_B4FP99 Harpin-induced protein n=1 Tax=Zea mays RepID=B4FP99_MAIZE | AT2G01080.1\|Symbols:\|Late embryogenesis abundant (LEA) hydroxyproline-rich glycoprotein family\|chr2:78038-79176 FORWARD LENGTH=231 |
| Glyma15g05430 | Glyma.15G048900 | Glyma.08G183900 | UniRef100_G7IL66 COP9 signalosome complex subunit n=1 Tax=Medicago truncatula RepID=G7IL66_MEDTR | AT4G14110.1\|Symbols:COP9, CSN8, FUS7, EMB143\|COP9 signalosome, subunit CSN8\|chr4:8133049-8134867 REVERSE LENGTH=197 |
| Glyma15g05440 | Glyma.15G049000 | Glyma.08G183600 | UniRef100_A4ZGU1 Transcription factor bZIP10 n=1 Tax=Glycine max RepID=A4ZGU1_SOYBN | AT5G44080.1\|Symbols:\|Basic-leucine zipper (bZIP) transcription factor family protein\|chr5:17738787-17739734 REVERSE LENGTH=315 |
| Glyma15g05450 | Glyma.15G049100 | - | UniRef100_G7IL69 Deacetylvindoline O-acetyltransferase n=1 Tax=Medicago truncatula RepID=G7IL69_MEDTR | AT3G26040.1\|Symbols:\|HXXXD-type acyl-transferase family protein\|chr3:9519741-9521069 FORWARD LENGTH=442 |
| Glyma15g05470 | Glyma.15G049200 | Glyma.08G183500 | UniRef100_G7K633 Protein RUPTURED POLLEN GRAIN n=1 Tax=Medicago truncatula RepID=G7K633_MEDTR | AT5G13170.1\|Symbols:SAG29, SWEET15, AtSWEET15\|senescence-associated gene 29\|chr5:4181331-4183171 REVERSE LENGTH=292 |
| Glyma15g05480 | Glyma.15G049300 | Glyma.08G183400 | UniRef100_Q9SLU9 F19C14.4 protein n=1 Tax=Arabidopsis thaliana RepID=Q9SLU9_ARATH | AT1G09980.1\|Symbols:\|Putative serine esterase family protein\|chr1:3256541-3260866 REVERSE LENGTH=802 |
| Glyma15g05490 | Glyma.15G049500 | - | UniRef100_G7IL74 Tubby-like F-box protein n=1 Tax=Medicago truncatula RepID=G7IL74_MEDTR | AT1G25280.1\|Symbols:AtTLP10, TLP10\|tubby like protein 10\|chr1:8864961-8866608 FORWARD LENGTH=445 |
| Glyma15g05510 | Glyma.15G049600 | Glyma.08G183000 | NA | AT1G25275.3 response to karrikin; - 531; Viruses - 0; Other Eukaryotes - 9610 (source: NCBI BLink).\|chr1:8860719-8861156 FORWARD LENGTH=85 |
| Glyma15g05520 | Glyma.15G049700 | Glyma.08G182900 | UniRef100_G7IL77 Auxin-induced protein 5NG4 n=1 Tax=Medicago truncatula RepID=G7IL77_MEDTR | AT1G25270.1\|Symbols:\|nodulin MtN21 /EamA-like transporter family protein\|chr1:8857726-8859909 FORWARD LENGTH=355 |
| Glyma15g05530 | Glyma.15G049800 | Glyma.08G182700 | UniRef100_G7IL80 Auxin-induced protein 5NG4 n=1 Tax=Medicago truncatula RepID=G7IL80_MEDTR | AT1G68170.1\|Symbols:\|nodulin MtN21 /EamA-like transporter family protein\|chr1:25551925-25554258 FORWARD LENGTH=356 |
| Glyma15g05540 | Glyma.15G049900 | Glyma.08G182500 | UniRef100_G7IL80 Auxin-induced protein 5NG4 n=1 Tax=Medicago truncatula RepID=G7IL80_MEDTR | AT1G25270.1\|Symbols:\|nodulin MtN21 /EamA-like transporter family protein\|chr1:8857726-8859909 FORWARD LENGTH=355 |
| Glyma15g05551 | - | Glyma.08G182400 | UniRef100_Q2KMJ4 EKN n=1 Tax=Glycine max RepID=Q2KMJ4_SOYBN | NA |
| Glyma15g05560 | Glyma.15G050100 | Glyma.08G182300 | UniRef100_G7IL84 Cytosolic fructose-1 6-bisphosphatase n=1 Tax=Medicago truncatula RepID=G7IL84_MEDTR | AT1G43670.1\|Symbols:\|Inositol monophosphatase family protein\|chr1:16468184-16470347 FORWARD LENGTH=341 |
| Glyma15g05570 | Glyma.15G050200 | Glyma.08G182200 | UniRef100_V5N8J4 Actin-101-like n=1 Tax=Phaseolus vulgaris RepID=V5N8J4_PHAVU | AT3G12110.1\|Symbols:ACT11\|actin-11\|chr3:3858116-3859609 FORWARD LENGTH=377 |
| Glyma15g05580 | Glyma.15G050300 | Glyma.08G182100 | UniRef100_O48923 Cytochrome P450 71D10 n=1 Tax=Glycine max RepID=C71DA_SOYBN | AT3G26330.1\|Symbols:CYP71B37\|cytochrome P450, family 71, subfamily B, polypeptide 37\|chr3:9646873-9648536 REVERSE LENGTH=500 |
| Glyma15g05610 | - | - |  |  |
| Glyma15g05620 | Glyma.15G050500 | - | UniRef100_C6T1W4 Cytochrome b-c1 complex subunit 6 n=1 Tax=Glycine max RepID=C6T1W4_SOYBN | AT1G15120.1\|Symbols:\|Ubiquinol-cytochrome C reductase hinge protein\|chr1:5203091-5203897 FORWARD LENGTH=69 |
| Glyma15g05630 | Glyma.15G050600 | - | UniRef100_G7IL92 Histidine decarboxylase n=1 Tax=Medicago truncatula RepID=G7IL92_MEDTR | AT1G43710.1\|Symbols:emb1075\|Pyridoxal phosphate (PLP)-dependent transferases superfamily protein\|chr1:16486534-16488298 REVERSE LENGTH=482 |
