# Supplementary figures and images for: Molecular mapping and genomics of soybean seed protein: a review and perspective for the future
Source: Theor Appl Genet. 2017 Aug 11;130(10):1975–91. doi: 10.1007/s00122-017-2955-8 (PMC5606949; doi:10.1007/s00122-017-2955-8)

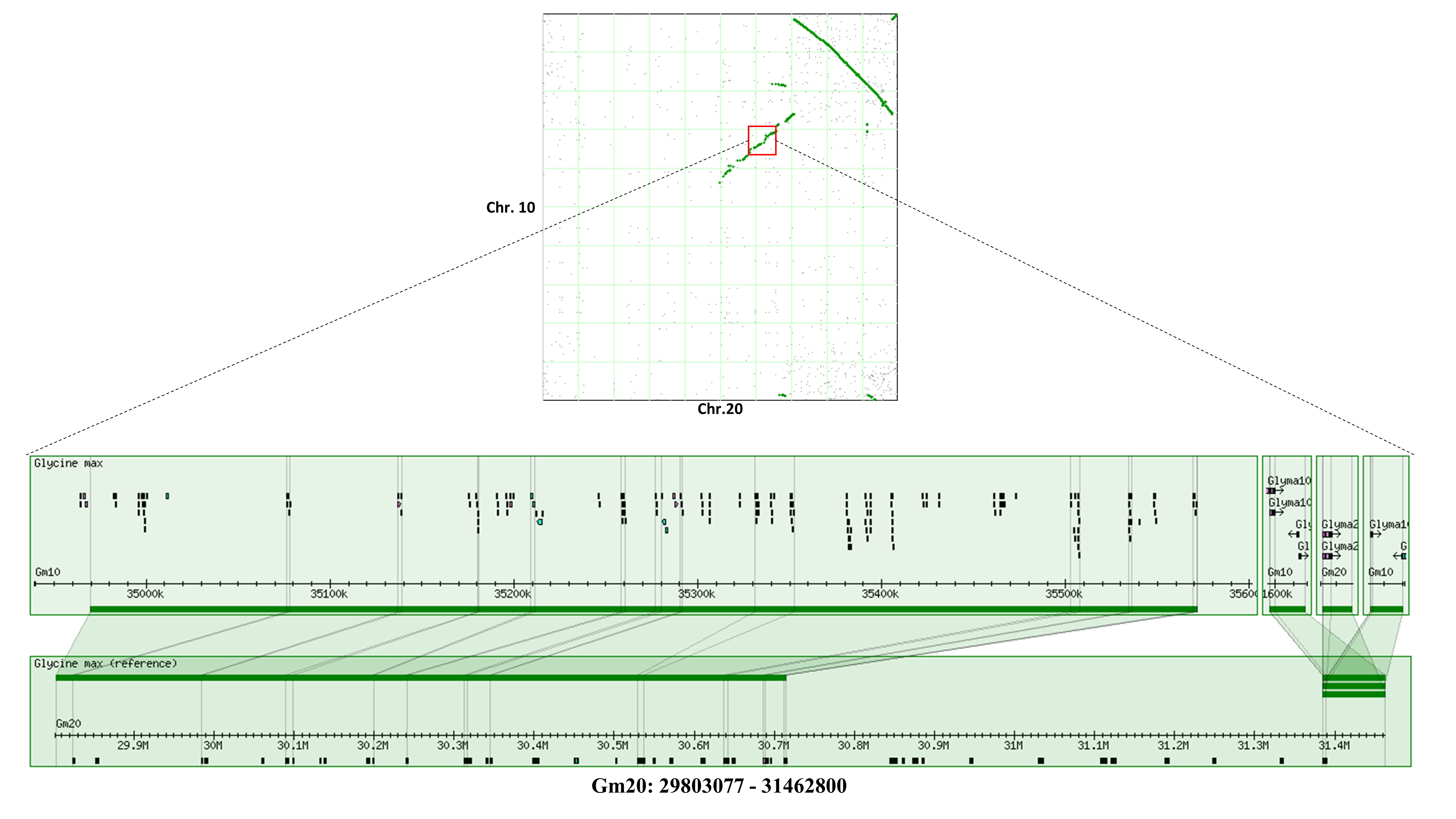

Supplement: Supplementary file 5 — Supplementary Figure 1: Syntenic analysis of major protein QTL on Chr. 20 (Gm20: 29803077–31462800) (JPEG 1461 kb) [file 122_2017_2955_MOESM5_ESM.jpg]
